# Norisoboldine, a natural AhR agonist, promotes Treg differentiation and attenuates colitis via targeting glycolysis and subsequent NAD^+^/SIRT1/SUV39H1/H3K9me3 signaling pathway

**DOI:** 10.1038/s41419-018-0297-3

**Published:** 2018-02-15

**Authors:** Qi Lv, Kai Wang, Simiao Qiao, Ling Yang, Yirong Xin, Yue Dai, Zhifeng Wei

**Affiliations:** Department of Pharmacology of Chinese Materia Medica, School of Traditional Chinese Pharmacy, China Pharmaceutical University, 24 Tong Jia Xiang, Nanjing, 210009 China

## Abstract

Norisoboldine (NOR), a natural aryl hydrocarbon receptor (AhR) agonist, has been demonstrated to attenuate ulcerative colitis (UC) and induce the generation of Treg cells. Under UC condition, hypoxia widely exists in colonic mucosa, and secondary changes of microRNAs (miRs) expressions and glycolysis contribute to Treg differentiation. At present, we worked for exploring the deep mechanisms for NOR-promoted Treg differentiation in hypoxia and its subsequent anti-UC action from the angle of AhR/miR or AhR/glycolysis axis. Results showed that NOR promoted Treg differentiation in hypoxia and the effect was stronger relative to normoxia. It activated AhR in CD4^+^ T cells under hypoxic microenvironment; CH223191 (a specific AhR antagonist) and siAhR-3 abolished NOR-promoted Treg differentiation. Furthermore, the progress of glycolysis, levels of Glut1 and HK2, and expression of *miR-31* rather than *miR-219* and *miR-490* in CD4^+^ T cells were downregulated by NOR treatment under hypoxic microenvironment. However, HK2 plasmid but not *miR-31* mimic significantly interfered NOR-enhanced Treg polarization. In addition, NOR reduced NAD^+^ and SIRT1 levels, facilitated the ubiquitin-proteasomal degradation of SUV39H1 protein, and inhibited the enrichment of H3K9me3 at −1, 201 to −1,500 region of Foxp3 promoter in CD4^+^ T cells under hypoxic microenvironment, which was weakened by HK2 plasmid, CH223191, and siAhR-3. Finally, the correlation between NOR-mediated activation of AhR, repression of glycolysis, regulation of NAD^+^/SIRT1/SUV39H1/H3K9me3 signals, induction of Treg cells, and remission of colitis was confirmed in mice with DSS-induced colitis by using CH223191 and HK2 plasmid. In conclusion, NOR promoted Treg differentiation and then alleviated the development of colitis by regulating AhR/glycolysis axis and subsequent NAD^+^/SIRT1/SUV39H1/H3K9me3 signaling pathway.

## Introduction

Regulatory T (Treg) cells are a unique subpopulation of CD4^+^ T cells, which have pivotal roles in maintenance of immune tolerance and prevention of autoimmunity against self-antigens. Treg cells can inhibit the proliferation and activation of T-effector (Teff) cells by cell–cell contact or secretion of transforming growth factor (TGF)-β, interleukin (IL)-10, granzyme, and perforin^1,2^. The deficiency of Treg cells has been linked to the occurrence and development of multiple autoimmune diseases in animals and humans, and adoptive transfer of Treg cells shows opposite effect. Therefore, boosting numbers of Treg cells is likely to be an effective strategy for the treatment of immune-related diseases including ulcerative colitis (UC), experimental autoimmune encephalomyelitis, etc.

The detailed mechanisms for Treg differentiation are still obscure and recent evidences suggest that hypoxia has an important role^3^. In response to hypoxia, the expressions of microRNAs (miRs) change and glycolytic switch occurs. Under hypoxic microenvironment, the expression of *miR-31* in dendritic cells (DCs) is elevated and the expression of *miR-1296* in hepatocellular carcinoma tissue is reduced^4,5^. Notably, *miR-212/132* cluster knockout mice show high percentage of IL-10-producing CD4^+^ T cells in colons^6^. In addition, *miR-31*, *miR-219*, and *miR-490* can bind with 3′-untranslated region of Foxp3 gene to regulate Treg differentiation^7^. Similarly, hypoxia contributes to switching the metabolism from oxidative phosphorylation to aerobic glycolysis in multiple kinds of cells, evidenced by increased production of metabolic acids^8^. 3-Bromopyruvate, a specific inhibitor of glycolysis, significantly decreases the arthritis scores of SKG mice by inducing Treg cells generation^9^. In parallel, 2-deoxy-d-glucose promotes the expression of Foxp3 under Treg -polarization condition^10^.

UC is a chronic inflammatory disorder of the colonic mucosa, which starts in the rectum and generally extends proximally in a continuous manner through part of, or the entire colon^11^. A robust hypoxia happens, because that profound neutrophils and macrophages infiltrating in colons require abundant oxygen to maintain growth, proliferation, apoptosis, and die^12^. Karhausen and colleagues.^13^ report the presence of hypoxia in colons by using 2-(2-nitro-1H-imidazol-1yl)-N-(2, 2, 3, 3,-pentafluoropropyl) acetamide to measure tissue oxygenation of colitis mice; Choi and colleagues.^14^ demonstrate that inflammatory hypoxia is observed in colons of mice with chronic colitis. Norisoboldine (NOR), the primary isoquinoline alkaloid of Radix Linderae, possesses well ability to activate aryl hydrocarbon receptor (AhR)^15^. In addition, it can effectively inhibit systemic inflammation in rats with adjuvant-induced arthritis or collagen-induced arthritis through a gut-dependent manner^16^. Subsequently, we demonstrate that NOR significantly alleviates colitis in dextran sulfate sodium (DSS)-induced mice and upregulates percentages of Treg cells in colons^17^. However, the detailed mechanisms are still enigmatic and need further investigation. At present, we explored the mechanisms for NOR-promoted Treg differentiation and subsequent anti-UC effect from the angle of miRs and glycolysis in hypoxia.

## Results

### NOR promotes the differentiation of Treg cells in hypoxia

In our previous study, NOR has been demonstrated to alleviate colitis in mice, which was accompanied with elevated percentages of Treg cells in colons^17^. However, the detailed mechanisms remain unknown. Recently, articles indicate that hypoxia widely exists in colonic mucosa and contribute to the differentiation of Treg cells^12,18^. Therefore, we first detected the effect of NOR on Treg differentiation in hypoxia.

**Fig. 1:**
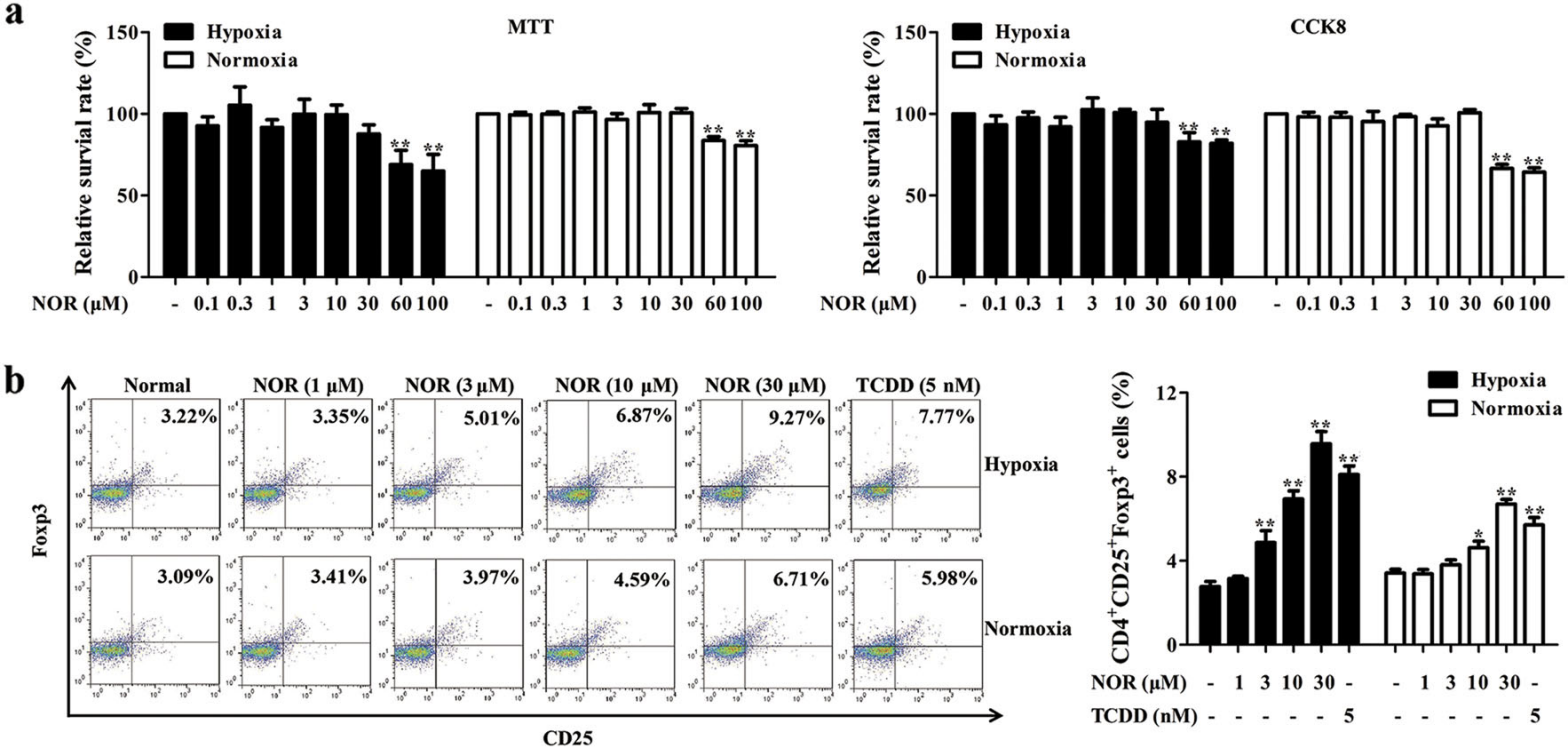
NOR promotes Treg differentiation under hypoxic and normoxic microenvironment. **a** CD4^*+*^ T cells were cultured with NOR (0.1, 0.3, 1, 3, 10, 30, 60, 100 μM) in hypoxia or normoxia for 72 h and cell viability was analyzed by MTT and CCK-8. **b** CD4^+^ T cells were cultured with anti-CD3/CD28 (2 µg/mL), NOR (1, 3, 10, 30 μM), and TCDD (5 nM) in hypoxia or normoxia for 72 h, and frequencies of Treg cells were analyzed by flow cytometry. Data were expressed as means ± SEM of three independent experiments. ^*^P < 0.05, ^**^P < 0.01 vs. Normal group

To exclude the interference of cytotoxicity on the action of NOR, viability of CD4^+^ T cells was tested. At the concentration below 60 μM, NOR did not exhibit obvious cytotoxicity of CD4^+^ T cells (Fig. 1a). Results of flow cytometry analysis revealed that the minimum effective concentration of NOR-promoted Treg differentiation in hypoxia was 3 μM, whereas in normoxia it was 10 μM. Of note, NOR (30 μM) increased the frequencies of Treg cells to 9.27% in hypoxia and 6.71% in normoxia (Fig. 1b). In parallel, similar results were observed on levels of Foxp3 and IL-10 (Supplementary Figure 1a and b).

The findings suggested that NOR could promote Treg differentiation in hypoxia and normoxia. The minimum effective concentration was lower and the action was stronger at the same concentration in hypoxia than normoxia. Subsequently, mechanisms for NOR-promoted Treg differentiation in hypoxia were explored.

### NOR induces the activation of AhR in hypoxia

Data indicate that AhR is favorable for Treg differentiation and NOR has been identified as a possible AhR agonist^15,19^. Therefore, the participation of AhR in NOR-mediated promotion of Treg differentiation under hypoxic microenvironment was determined. 2, 3, 7, 8-Tetrachlorodibenzo-p-dioxin (TCDD) is a classical AhR agonist and can promote Treg differentiation in normoxia. To compare and find out the similarities and differences between NOR and classical AhR agonist in Treg differentiation under hypoxic microenvironment, TCDD was adopted as a positive drug.

**Fig. 2:**
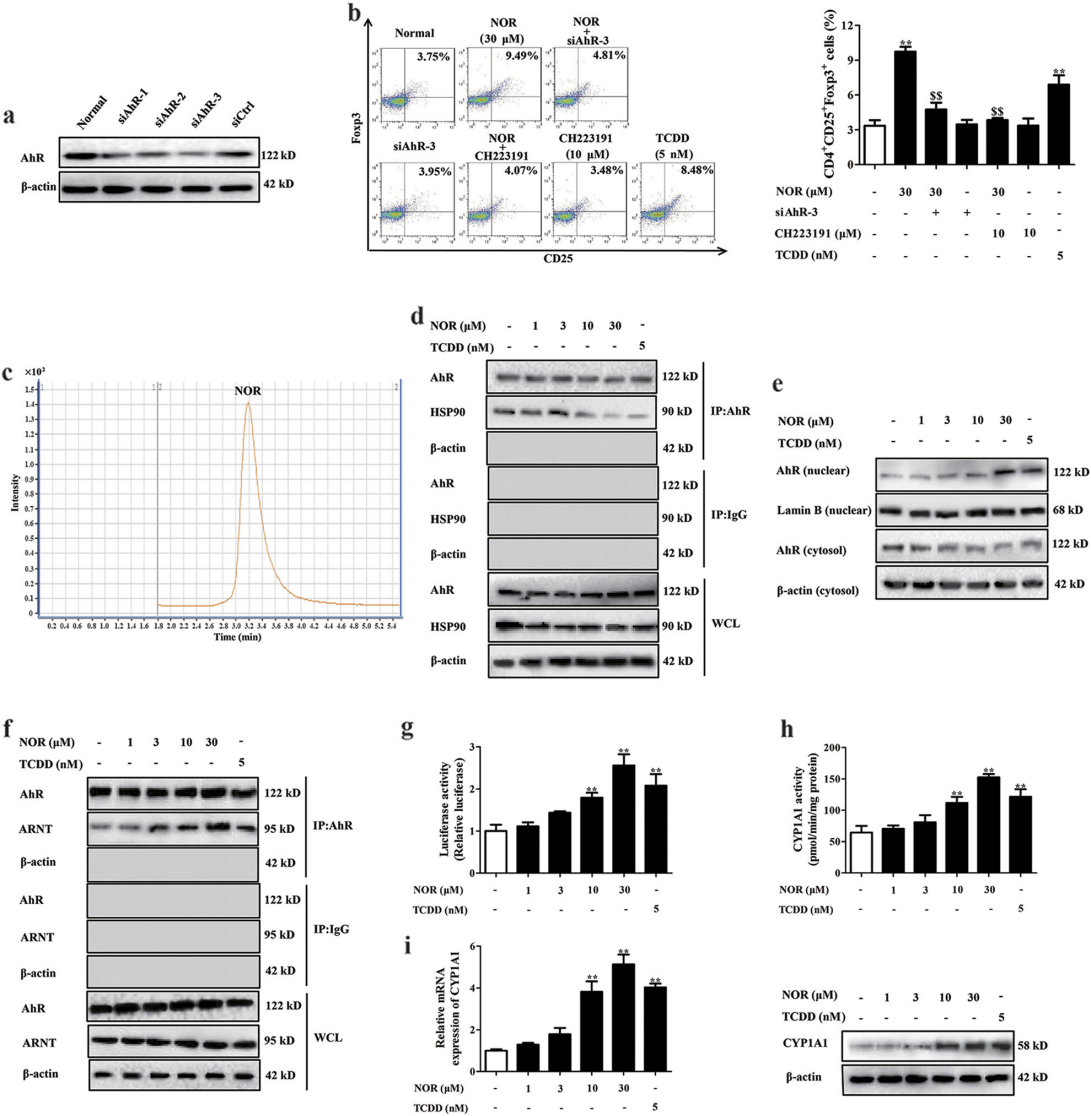
NOR activates AhR in CD4^+^ T cells under hypoxic microenvironment. **a** CD4^+^ T cells were transfected with siAhR1-3 or siCtrl, and AhR expression was analyzed by western blot. **b** CD4^+^ T cells were pretreated with CH223191 (10 μM) for 2 h or transfected with siAhR-3, followed with anti-CD3/CD28 (2 µg/ml), NOR (1, 3, 10, 30 μM), and TCDD (5 nM) in hypoxia for 72 h, and frequencies of Treg cells were analyzed by flow cytometry. **c** CD4^+^ T cells were cultured with anti-CD3/CD28 (2 µg/ml), NOR (30 μM) in hypoxia for 4 h, and the uptake of NOR was analyzed by LC-MS. **d**–**i** CD4^+^ T cells were cultured with anti-CD3/CD28 (2 µg/ml), NOR (1, 3, 10, 30 μM), and TCDD (5 nM) in hypoxia for 24 h. The dissociation of AhR/HSP90 complexes was analyzed by co-immunoprecipitation **d**; the protein level of AhR in cytosol and nuclear was analyzed by western blotting **e**; the formation of AhR/ARNT complexes was analyzed by co-immunoprecipitation **f**; the XRE-luciferase reporter gene activity was analyzed by kits **g**; the EROD activity, mRNA expression, and protein level of CYP1A1 were analyzed by kits, Q-PCR, and western blotting, respectively **h**, **i**. Data were expressed as means ± SEM of three independent experiments. ^**^P < 0.01 vs. Normal group; ^$$^*P* < 0.01 vs. NOR (30 μM) group

In Fig. 2a, all the three pairs of siAhR inhibited AhR expression and siAhR-3 showed the best efficiency. Furthermore, NOR (30 μM) significantly enriched Treg differentiation in hypoxia, siAhR-3 and CH223191 (10 μM) dramatically prevented the action (Fig. 2b). Consistently, siAhR-3 and CH223191 significantly weakened NOR-induced expressions of Foxp3 and IL-10 in CD4^+^ T cells under hypoxic microenvironment (Supplementary Figure 2a and b).

The effect of NOR on AhR activation in CD4^+^ T cells under hypoxic microenvironment has not been demonstrated and the following experiments were performed. Results of liquid chromatography–mass spectrometry (LC–MS) showed that NOR could enter into cytoplasm of CD4^+^ T cells (Fig. 2c). Moreover, NOR (10, 30 μM) and TCDD (5 nM) facilitated the disassociation of HSP90/AhR complexes, the nuclear translocation of AhR, and the formation of AhR/ARNT complexes. Lastly, the activity of xenobiotic response element (XRE)-luciferase reporter gene, expressions and enzyme activity of CYP1A1 were also elevated (Fig. 2d–i). These results indicated that NOR drove Treg cells abundance in an AhR-dependent manner under hypoxic microenvironment.

### NOR-promoted Treg differentiation is independent of miRs in hypoxia

The miRs represent a class of evolutionarily conserved regulatory RNAs, which modulate gene expressions including Foxp3^20^. In hypoxia, expressions of multiple miRs change and the miRs function as key mediators for the action of AhR activation. Furthermore, 3, 3'-diindolylmethane (DIM) and indole-3-carbinol (I3C), the classical AhR agonists, promote Treg differentiation by targeting *miR-31*, *miR-219*, and *miR-490*^4,5,7^.

**Fig. 3:**
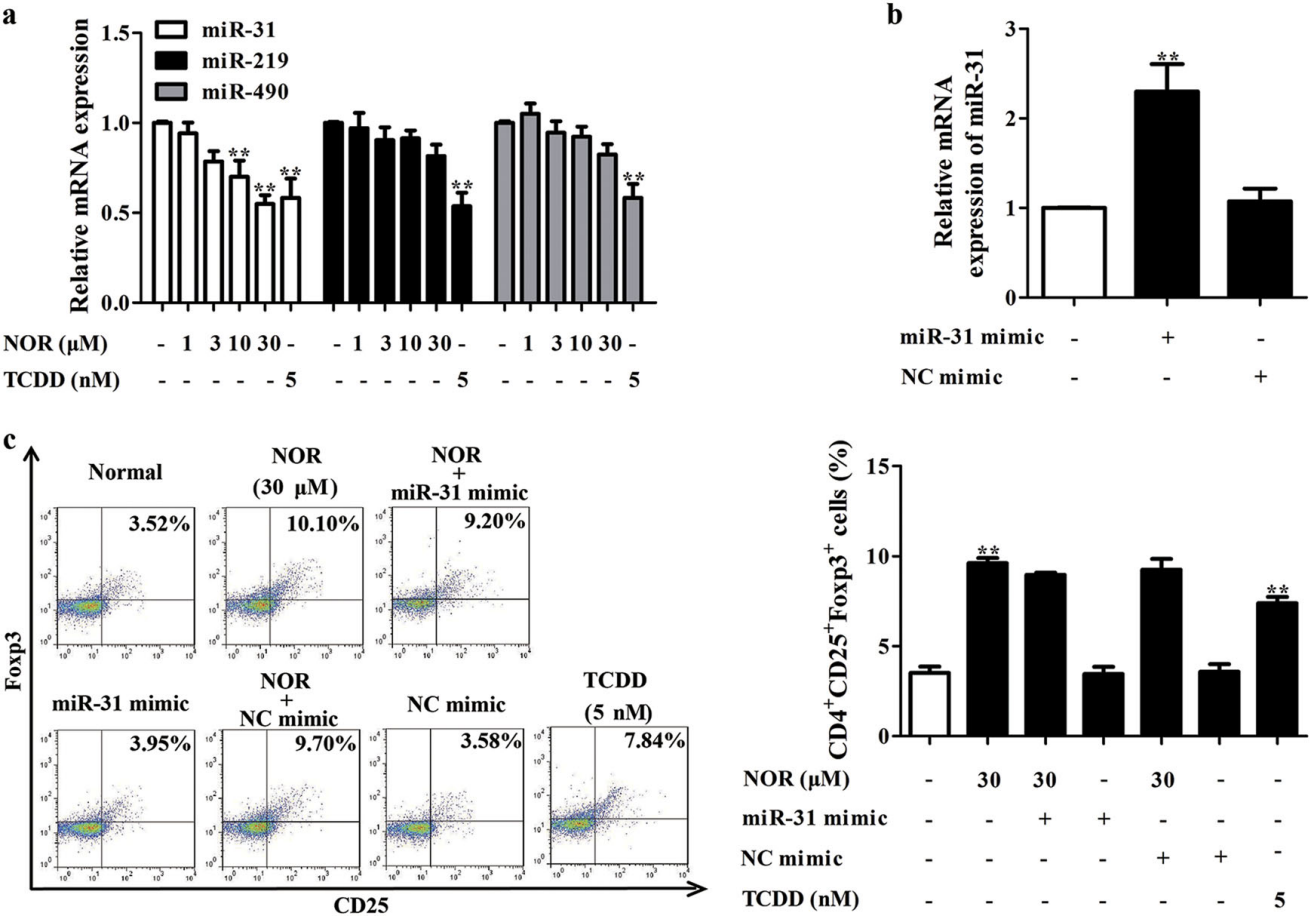
NOR-promoted Treg differentiation is independent of miRs under hypoxic microenvironment. **a** CD4^+^ T cells were cultured with anti-CD3/CD28 (2 µg/ml), NOR (1, 3, 10, 30 μM), and TCDD (5 nM) in hypoxia for 24 h. The mRNA expressions of *miR-31*, *miR-219*, and *miR-490* were analyzed by Q-PCR. **b** CD4^+^ T cells were transfected with *miR-31* or NC mimic and mRNA expression of *miR-31* was analyzed by Q-PCR. **c** CD4^+^ T cells were transfected with *miR-31* or NC mimic, followed with incubation of anti-CD3/CD28 (2 µg/ml), NOR (30 μM), and TCDD (5 nM) in hypoxia for 72 h, and frequencies of Treg cells were analyzed by flow cytometry. Data were expressed as means ± SEM of three independent experiments. ^**^P < 0.01 vs. Normal group

Thus, quantitative-PCR (Q-PCR) assay was performed to assess effect of NOR on mRNA expressions of *miR-31*, *miR-219*, and *miR-490* in CD4^+^ T cells. In Fig. 3a, NOR (10, 30 μM) significantly downregulated mRNA expression of *miR-31*, but not *miR-219* and *miR-490* in CD4^+^ T cells under hypoxic atmosphere. However, *miR-31* mimic showed little effect of NOR-promoted Treg differentiation (Fig. 3b, c), expressions of Foxp3 and IL-10 (Supplementary Figure 3a and b) in hypoxia. These interesting results indicated that NOR-induced generation of Treg cells in hypoxia was independent of *miR-31*.

### NOR enhances Treg polarization via a glycolysis-dependent manner in hypoxia

**Fig. 4:**
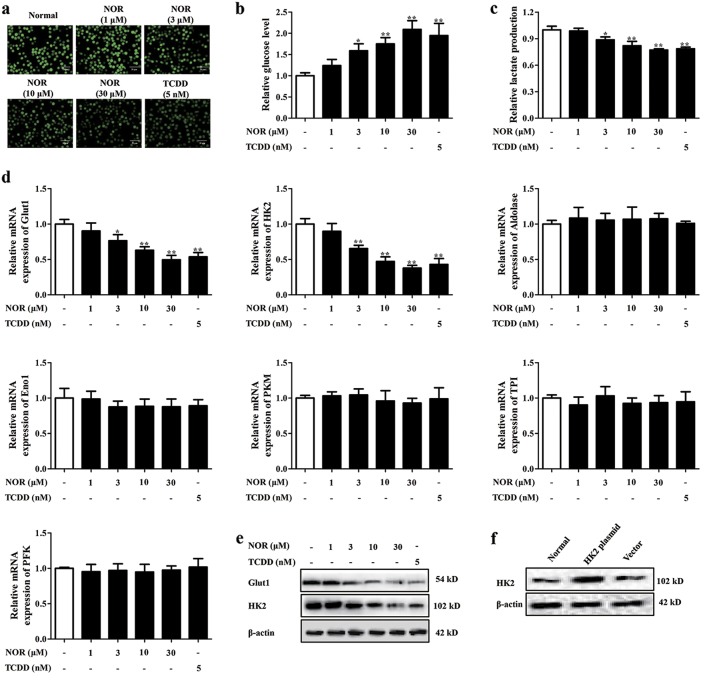

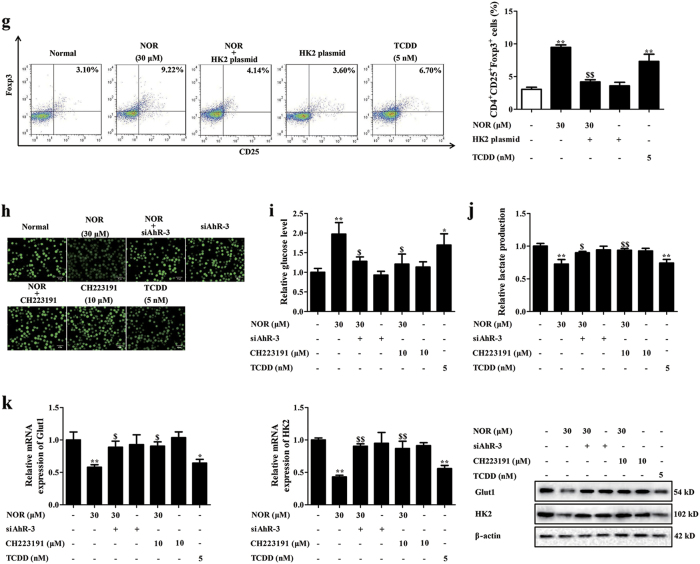
NOR enhances Treg polarization via a glycolysis-dependent manner under hypoxic microenvironment. **a**–**e** CD4^+^ T cells were cultured with anti-CD3/CD28 (2 µg/ml), NOR (1, 3, 10, 30 μM), and TCDD (5 nM) in hypoxia for 24 h. Glucose uptake was analyzed by immunofluorescence and the images were taken at × 200 magnification (scale bar: 50 μm) **a**; glucose consumption was analyzed by kits **b**; lactate production was analyzed by kits **c**; mRNA expressions of Glut1, HK2, Aldolase, Eno1, PKM, TPI, and PFK were analyzed by Q-PCR **d**; protein levels of Glut1 and HK2 were analyzed by western blotting **e**. **f** CD4^+^ T cells were transfected with HK2 plasmid or vector and protein level of HK2 was detected by western blotting. **g** CD4^+^ T cells were transfected with HK2 plasmid, followed with incubation of anti-CD3/CD28 (2 µg/ml), NOR (30 μM), and TCDD (5 nM) in hypoxia for 72 h, and frequencies of Treg cells were analyzed by flow cytometry. **h**–**k** CD4^+^ T cells were pretreated with CH223191 (10 µM) for 2 h or transfected with siAhR-3, followed with incubation of anti-CD3/CD28 (2 µg/ml), NOR (30 μM), and TCDD (5 nM) in hypoxia for 24 h. Glucose uptake was analyzed by immunofluorescence and the images were taken at × 200 magnification (scale bar: 50 μm) **h**; glucose consumption was analyzed by kits **i**; lactate production was analyzed by kits **j**; mRNA and protein levels of HK2 and Glut1 were analyzed by Q-PCR and western blotting, respectively **k**. Data were expressed as means ± SEM of three independent experiments. ^*^P < 0.05, ^**^P < 0.01 vs. Normal group; ^$^*P* < 0.05, ^$$^*P* < 0.01 vs. NOR (30 μM) group

Glycolysis is a metabolic pathway that catabolizes glucose to induce the generation of lactate. Data reveal that glycolysis in cells is strengthened in hypoxia and blocking it promotes Treg differentiation^9,10^. Herein, effect of NOR on glycolysis in CD4^+^ T cells was measured. NOR (3, 10, 30 μM) and TCDD (5 nM) markedly restrained the uptake of 2-[N-(7-nitrobenz-2-oxa-1, 3-diazol-4-yl) amino]-2-deoxy-d-glucose (2-NBDG), consumption of glucose, and production of lactate in CD4^+^ T cells under hypoxic atmosphere (Fig. 4a–c). However, NOR (3, 10, 30 μM) and TCDD (5 nM) did not influence glycolysis in CD4^+^ T cells under normoxic atmosphere (Supplementary Figure 4a-c).

The progress of glycolysis is controlled by serials of enzymes, including hexokinase II (HK2), phosphoglucoisomerase, phosphofructokinase (PFK), glyceraldehyde 3-phosphatedehydrogenase (GAPDH), phosphaglycerate kinase, phosphoglycerate mutase, triosephosphate isomerase (TPI), enolase (Eno1), Aldolase, lactate dehydrogenase (LDH), and pyruvate kinase M (PKM). Notably, Glut1 is the selectively glucose transporter of T cells, HK2, PFK, and PKM are the rate-limiting enzymes in glycolysis^21^. Specially, expressions of TPI, Eno1, Aldolase, and PKM are increased in colonic mucosa of UC patients. To verify which enzyme contributes to NOR-depressed glycolysis in CD4^+^ T cells, the levels of Glut1, HK2, TPI, Eno1, Aldolase, PFK, and PKM were determined. NOR (3, 10, 30 μM) and TCDD (5 nM) gradually reduced mRNA and protein levels of Glut1 and HK2 in CD4^+^ T cells, and the reduction was more prominent on level of HK2 (Fig. 4d, e). Conversely, the expressions of Glut1, HK2, TPI, Eno1, Aldolase, PFK, and PKM were not affected by NOR (3, 10, 30 μM) and TCDD (5 nM) in normoxia (Supplementary Figure 4d and e).

Increasing evidence indicates that HK2 but not Glut1 is required for Treg differentiation in vitro and in vivo^9,10,21^. More importantly, NOR showed stronger reduction of HK2 level relative to Glut1. Therefore, HK2 plasmid was applied to verify the participation of glycolysis in NOR-mediated Treg polarization under hypoxic microenvironment. However, NOR-induced Treg differentiation was not affected by HK2 plasmid in normoxia (Supplementary Figure 4f–h). As expected, HK2 plasmid significantly weakened NOR (30 μM)-promoted Treg differentiation (Fig. 4f, g), expressions of Foxp3 and IL-10 in hypoxia (Supplementary Figure 5a and b). Further results showed that siAhR-3 and CH223191 (10 μM) almost completely restored NOR-inhibited glycolysis of CD4^+^ T cells in hypoxia (Fig. 4h–k).

Considering that HIF-1α ubiquitously expresses in hypoxia, HK2 and Glut1 are demonstrated as target genes of HIF-1α, the following experiments were performed, and NOR (1, 3, 10, 30 μM) and TCDD (5 nM) barely influenced the level of HIF-1α, whereas it remarkably downregulated the formation of HIF-1α/ARNT complexes in hypoxia (Supplementary Figure 6a and b). All these results might imply that NOR reduced expressions of HK2 and Glut1, and glycolysis by inhibiting the formation of HIF-1α/ARNT complexes in CD4^+^ T cells under hypoxic microenvironment.

### NOR decreases the levels of NAD^+^ and SIRT1 in hypoxia

**Fig. 5:**
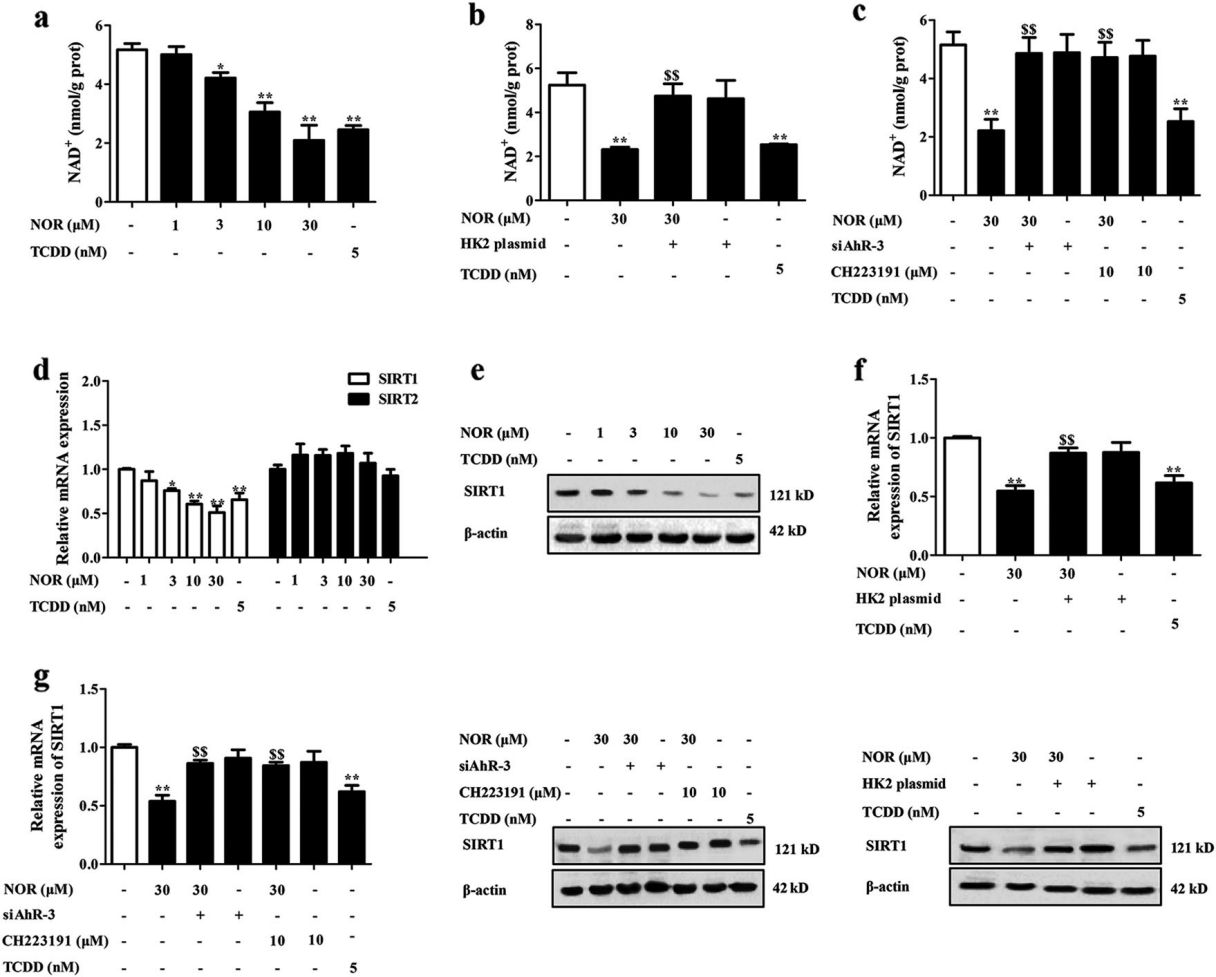
NOR decreases NAD^+^ and SIRT1 levels in CD4^+^ T cells under hypoxic microenvironment. **a** CD4^+^ T cells were cultured with anti-CD3/CD28 (2 µg/ml), NOR (1, 3, 10, 30 μM), and TCDD (5 nM) in hypoxia for 48 h, and NAD^+^ level was analyzed by using kits. **b**, **c** CD4^+^ T cells were pretreated with CH223191 (10 μM) for 2 h or transfected with HK2 plasmid/siAhR-3, followed with incubation of anti-CD3/CD28 (2 µg/ml), NOR (1, 3, 10, 30 μM), and TCDD (5 nM) in hypoxia for 48 h. NAD^+^ level was analyzed by using kits. **d**, **e** CD4^+^ T cells were cultured with anti-CD3/CD28 (2 µg/ml), NOR (1, 3, 10, 30 μM), and TCDD (5 nM) in hypoxia for 48 h. The mRNA levels of SIRT1 and 2 were analyzed by Q-PCR **d**; the protein level of SIRT1 was analyzed by western blotting **e**. **f**, **g** CD4^+^ T cells were pretreated with CH223191 (10 μM) for 2 h or transfected with HK2 plasmid/siAhR-3, followed with incubation of anti-CD3/CD28 (2 µg/ml), NOR (30 μM) and TCDD (5 nM) in hypoxia for 48 h. The mRNA and protein levels of SIRT1 were analyzed by Q-PCR and western blotting, respectively. Data were expressed as means ± SEM of three independent experiments. ^*^P < 0.05, ^**^P < 0.01 vs. Normal group; ^$^*P* < 0.05, ^$$^*P* < 0.01 vs. NOR (30 μM) group

Then, the precise mechanisms responsible for NOR-promoted Treg differentiation after repression of glycolysis were explored. It is well established that nicotinamide adenine dinucleotide (NAD^+^) level increases accompanied with the progress of glycolysis and drives the shift of Treg cells toward Th17 cells^22,23^. Here, NOR (3, 10, 30 μM) and TCDD (5 nM) significantly decreased NAD^+^ level in CD4^+^ T cells and the effect was diminished by HK2 plasmid, CH223191 (10 μM), and siAhR-3 (Fig. 5a–c).

Importantly, NAD^+^ has been demonstrated as the substrate and agonist of sirtuins (SIRTs). SIRT1 and SIRT2 are localized at the nucleus and cytoplasm, and possess the deacetylase activity^24^. Considering that the process of glycolysis occurs in the cytoplasm, mRNA expressions of SIRT1 and SIRT2 were detected. NOR (3, 10, 30 μM) and TCDD (5 nM) significantly decreased the mRNA expression of SIRT1 in CD4^+^ T cells under hypoxic microenvironment, but not SIRT2 (Fig. 5d). Consistently, the protein level of SIRT1 was also markedly reduced (Fig. 5e). Furthermore, NOR-decreased SIRT1 expression in CD4^+^ T cells under hypoxic microenvironment was recused by HK2 plasmid, CH223191 (10 μM), and siAhR-3 (Fig. 5f, g).

### NOR facilitates the ubiquitin-proteasomal degradation of SUV39H1 in hypoxia

Data indicate that SIRT1 drives the abundance of Treg cells by directly regulating the acetylation level of Foxp3 protein^25^. However, NOR elevated both protein and mRNA levels of Foxp3 in CD4^+^ T cells. Therefore, we supposed that SIRT1 had an indirect role in NOR-promoted Treg differentiation. Multiple studies demonstrate that SIRT1 can directly regulate the expressions of histone methyltransferases (KMTs) and histone demethylases (KDMs), which are altered in hypoxia, including MLL1, G9a, SUV39H1, JMJD3, and EZH2^26–33^. Therefore, effects of NOR and TCDD on protein levels of MLL1, SUV39H1, G9a, EZH2, and JMJD3 were detected.

**Fig. 6:**
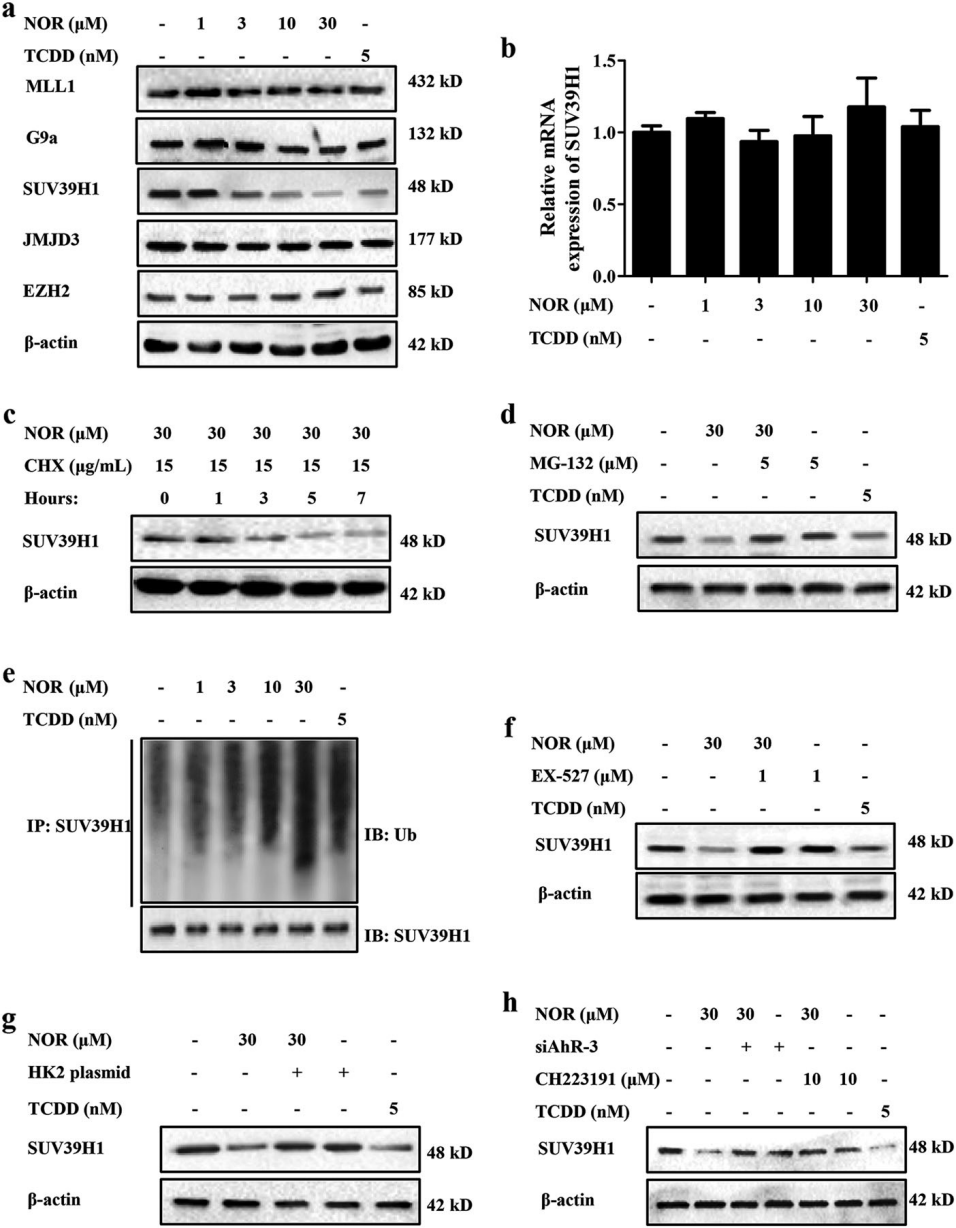
NOR facilitates the ubiquitin-proteasomal degradation of SUV39H1 protein in CD4^+^ T cells under hypoxic microenvironment. **a**–**c** CD4^+^ T cells were cultured with anti-CD3/CD28 (2 µg/ml), NOR (1, 3, 10, 30 μM), and TCDD (5 nM) in hypoxia for 48 h. The protein levels of MLL1, G9a, SUV39H1, JMJD3, and EZH2 were analyzed by western blotting **a**; the mRNA level of SUV39H1 was analyzed by Q-PCR **b**; the turnover of SUV39H1 protein was analyzed by pulse-chase experiment **c**. **d** CD4^+^ T cells were cultured with anti-CD3/CD28 (2 µg/ml), MG132 (5 μM), NOR (30 μM), and TCDD (5 nM) in hypoxia for 48 h, and the protein level of SUV39H1 was analyzed by western blotting. **e** CD4^+^ T cells were cultured with anti-CD3/CD28 (2 µg/ml), NOR (1, 3, 10, 30 μM), and TCDD (5 nM) in hypoxia for 48 h, and the ubiquitination level of SUV39H1 was analyzed. **f**–**h** CD4^+^ T cells were pretreated with EX-527 (1 μM)/CH223191 (10 μM) for 2 h or transfected with HK2 plasmid/siAhR-3, followed with incubation of anti-CD3/CD28 (2 µg/ml), NOR (30 μM), and (5 nM) in hypoxia for 48 h. The protein level of SUV39H1 was analyzed by western blotting. Data were expressed as means ± SEM of three independent experiments

NOR (3, 10, 30 μM) and TCDD (5 nM) remarkably reduced the protein level of SUV39H1, whereas the others remained unchanged (Fig. 6a). Next, to further confirm the impact of NOR and TCDD on SUV39H1, Q-PCR assay was adopted. Unfortunately, NOR (3, 10, 30 μM) and TCDD (5 nM) scarcely influenced mRNA expression of SUV39H1 (Fig. 6b). However, the further pulse-chase experiment indicated that NOR facilitated the turnover of SUV39H1 protein (Fig. 6c). These findings suggested that NOR regulated the expression of SUV39H1 by a posttranscriptional mechanism.

The degradation of SUV39H1 is via the ubiquitin-proteasomal pathway and MG-132 (a proteasome inhibitor) is employed. As shown in Fig. 6d, MG-132 (5 μM) restored NOR-reduced protein level of SUV39H1 in CD4^+^ T cells under hypoxic microenvironment. Furthermore, NOR (3, 10, 30 μM) and TCDD (5 nM) significantly increased the polyubiquitination level of SUV39H1 (Fig. 6e). In addition, EX-527 (a specific SIRT1 inhibitor; 1 μM), HK2 plasmid, CH223191 (10 μM), and siAhR-3 prevented NOR-reduced protein level of SUV39H1 in CD4^+^ T cells under hypoxic microenvironment (Fig. 6f–h).

### H3K9me3 modification has a crucial role in NOR-enhanced Treg polarization in hypoxia

**Fig. 7:**
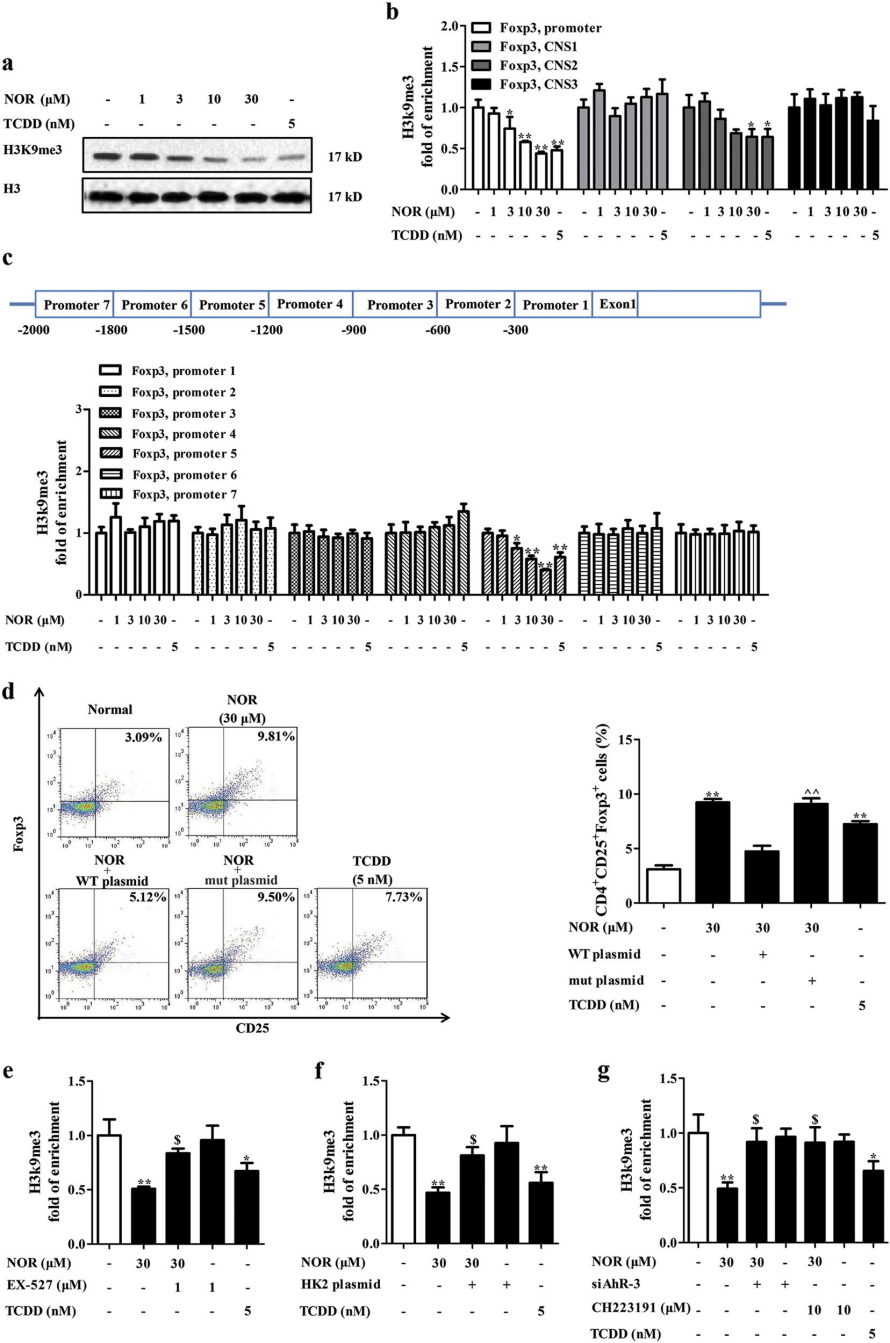
H3K9me3 modification has an important role in NOR-induced Treg differentiation under hypoxic microenvironment. **a**–**c** CD4^+^ T cells were cultured with anti-CD3/CD28 (2 µg/ml), NOR (1, 3, 10, 30 μM), and TCDD (5 nM) in hypoxia for 48 h. The global level of H3K9me3 was analyzed by western blotting **a**; the enrichment of H3K9me3 in Foxp3 promoter, CNS1, 2, and 3 regions was analyzed by ChIP **b**; the enrichment of H3K9me3 in − 1 to − 300 (Foxp3 promoter 1), − 301 to − 600 (Foxp3 promoter 2), − 601 to − 900 (Foxp3 promoter 3), − 901 to − 1,200 (Foxp3 promoter 4), − 1,201 to − 1,500 (Foxp3 promoter 5), − 1,501 to − 1,800 (Foxp3 promoter 6), and − 1,801 to − 2,000 (Foxp3 promoter 7) regions was analyzed by ChIP **c**. **d** CD4^+^ T cells were transfected with bacterial-contained wild-type Foxp3 promoter (WT) or − 1,201 to − 1,500 region deletion mutant (mut) plasmid, followed with incubation of anti-CD3/CD28 (2 µg/ml), NOR (30 μM), and TCDD (5 nM) in hypoxia for 72 h, and frequencies of Treg cells were analyzed by flow cytometry. **e**–**g** CD4^+^ T cells were pretreated with EX-527 (1 μM)/CH223191 (10 μM) for 2 h or transfected with HK2 plasmid/siAhR-3, followed with anti-CD3/CD28 (2 µg/ml), NOR (30 μM) in hypoxia for 48 h. The enrichment of H3K9me3 at − 1,201 to − 1,500 region of Foxp3 promoter was analyzed by ChIP. Data were expressed as means ± SEM of three independent experiments. ^*^P < 0.05, ^**^P < 0.01 vs. Normal group; ^$^*P* < 0.05 vs. NOR (30 μM) group. ^^^^*P* < 0.01 vs. NOR + WT plasmid group

SUV39H1 is a major member of histone KMTs and catalyzes the H3K9me3 modification, which is associated with transcription repression of Foxp3^34^. In Fig. 7a, NOR (3, 10, 30 μM) and TCDD (5 nM) significantly reduced the global level of H3K9me3 in CD4^+^ T cells under hypoxic microenvironment. Furthermore, histone modification at conserved non-coding sequences (CNSs) including CNS1, 2, and 3, and promoter of Foxp3 loci is important for the expression of Foxp3^35^. NOR and TCDD significantly decreased the enrichment of H3K9me3 at Foxp3 promoter and CNS2 regions, and the decrease was more prominent at Foxp3 promoter (Fig. 7b).

In order to investigate which motif at Foxp3 promoter was influenced by NOR and TCDD, seven pairs primers at Foxp3 promoter region were designed, and chromatin immunoprecipitation (ChIP) assay was performed. NOR (3, 10, 30 μM) and TCDD (5 nM) effectively reduced the H3K9me3 modification at − 1,201 to − 1,500 region of Foxp3 promoter (Fig. 7c). More importantly, significant differences existed in NOR-promoted Treg differentiation (Fig. 7d), expressions of Foxp3 and IL-10 (Supplementary Figure 7a and b) between cells transfected with wild-type Foxp3 promoter (WT) or − 1,201 to − 1,500 region deletion Foxp3 promoter mutant (mut) plasmid. Further studies indicated that NOR-reduced enrichment of H3K9me3 at − 1,201 to − 1,500 region of Foxp3 promoter was reversed by EX-527 (1 μM), HK2 plasmid, CH223191 (10 μM), and siAhR-3 (Fig. 7e–g).

### NOR drives Treg cells abundance to alleviate colitis in mice via modulating AhR/glycolysis axis and subsequent NAD^+^/SIRT1/SUV39H1/H3K9me3 signaling pathway

**Fig. 8:**
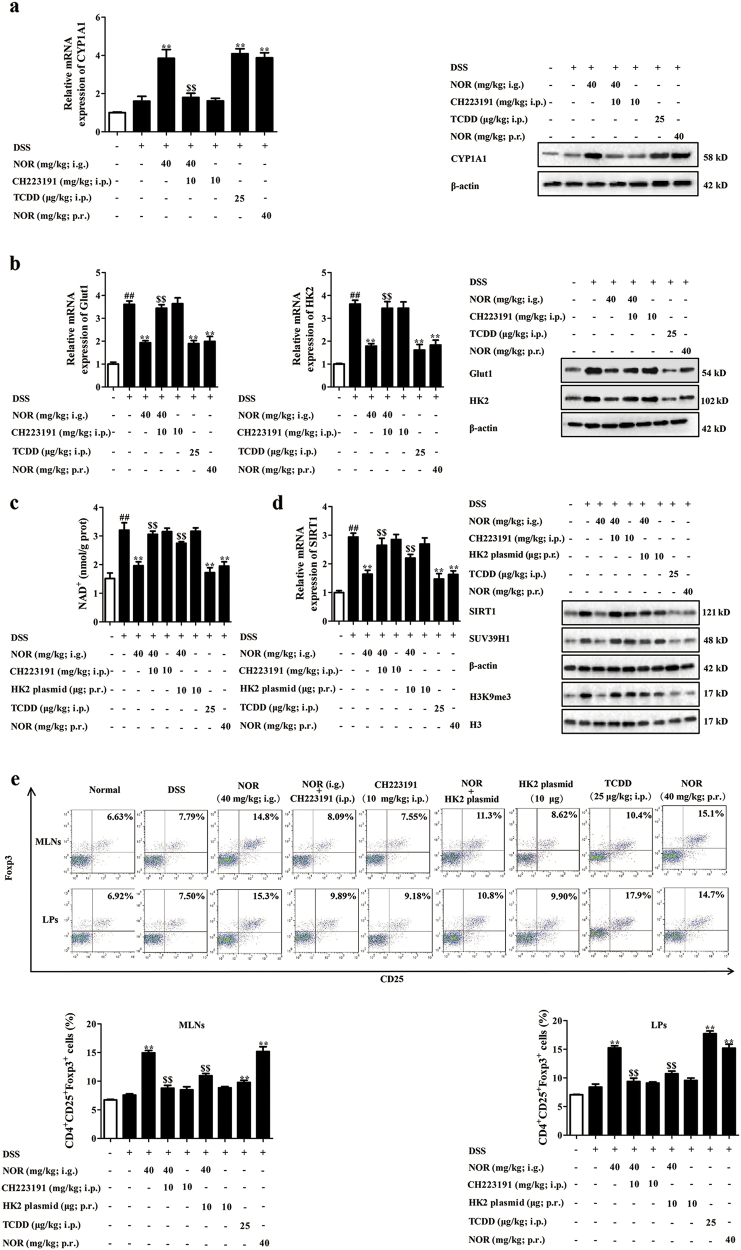

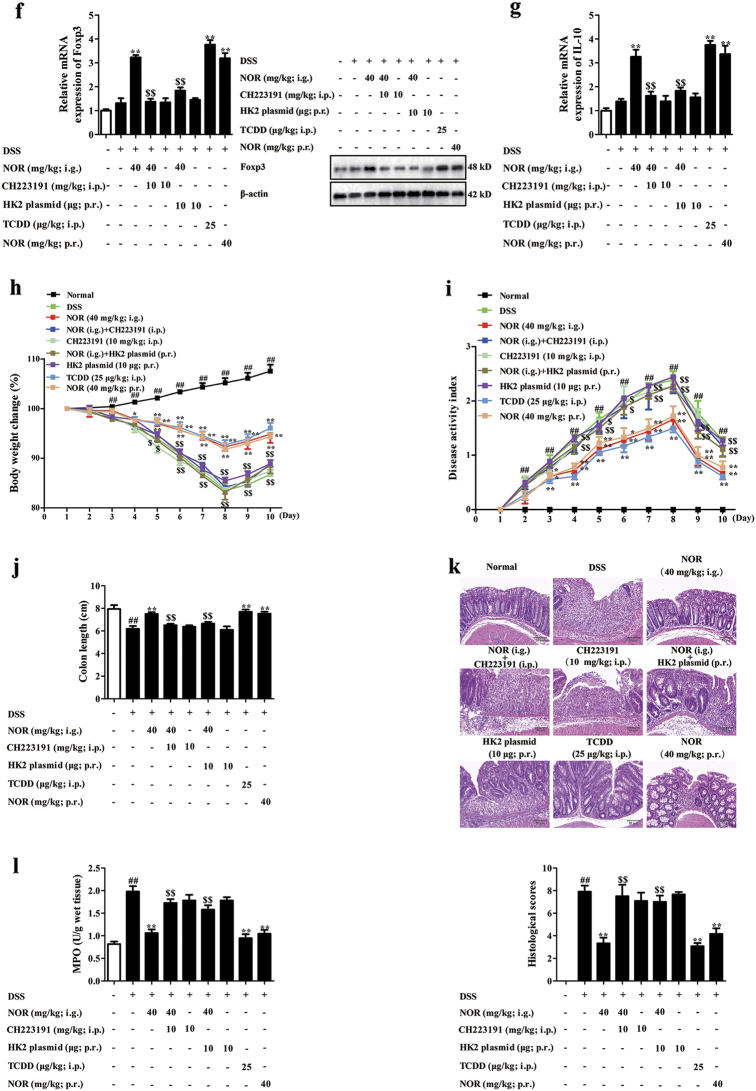

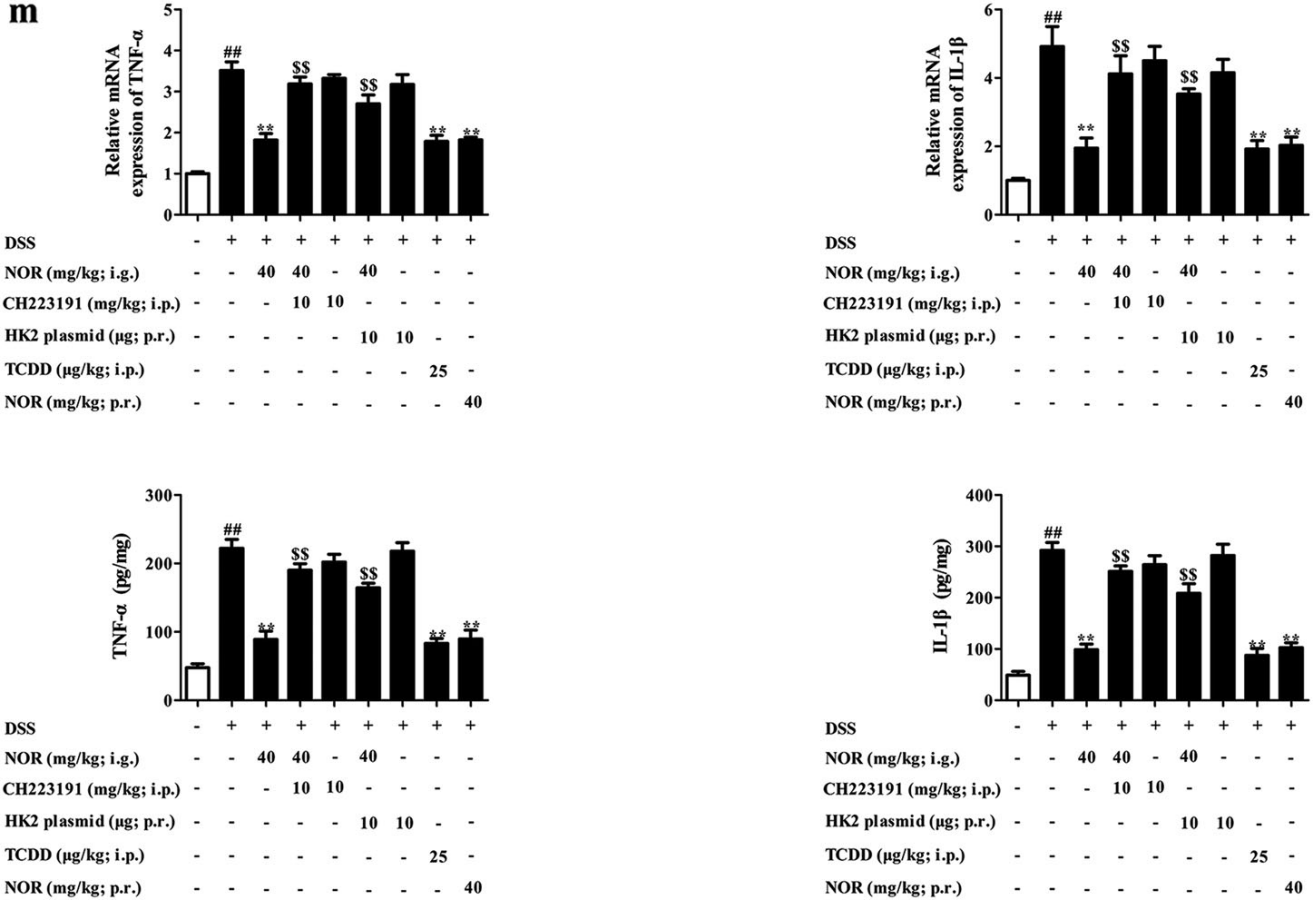
NOR drives Treg cells abundance to alleviate colitis in mice via modulating AhR/glycolysis axis and subsequent NAD^+^/SIRT1/SUV39H1/H3K9me3 signaling pathway. Mice were fed with 2.5% DSS for 7 days and followed by 3 days of drinking water alone. TCDD (25 µg/kg; i.p.) was administered once on day 1, whereas NOR (40 mg/kg; i.g. and p.r.), CH223191 (10 mg/kg; i.p.), and HK2 plasmid (10 µg; p.r.) were administered for 10 days. **a** The mRNA and protein levels of CYP1A1 in colons were analyzed by Q-PCR and western blotting, respectively. **b** The mRNA and protein levels of HK2 and Glut1 in colons were analyzed by Q-PCR and western blotting. **c** The level of NAD^+^ in colons was analyzed by kits. **d** The mRNA level of SIRT1 in colons was analyzed by Q-PCR; the protein levels of SIRT1, SUV39H1, and H3K9me3 in colons were analyzed by western blotting. **e** The frequencies of Treg cells in mesenteric lymph nodes (MLNs) and colonic lamina proprias (LPs) were analyzed by flow cytometry. **f** The mRNA and protein levels of Foxp3 in colons were analyzed by Q-PCR and western blotting, respectively. **g** The mRNA expression of IL-10 in colons was analyzed by Q-PCR. **h** Body weight change was analyzed. **i** Disease activity index (DAI) scores were calculated. **j** Colon length was analyzed. **k** Histopathological changes of colons were analyzed by H&E staining and the images were taken at × 200 magnification (scale bar: 50 μm). **l** Myeloperoxidase (MPO) activity was analyzed by kits. **m** The mRNA and protein levels of TNF-α and IL-1β were analyzed by Q-PCR and ELISA. Data were expressed as means ± SEM of six mice in each group. ^##^*P* < 0.01 vs. Normal group; ^*^P < 0.05 and ^**^P < 0.01 vs. DSS group; ^$^*P < *0.05 and ^$$^*P < *0.01 vs. NOR (40 mg/kg; i.g.) group

The effective form for the anti-colitis action of NOR was verified via oral (i.g.) and intra-rectal (p.r.) administration. On the whole, i.g. and p.r. administration of NOR (40 mg/kg) effectively ameliorated colitis in mice and elevated percentages of Treg cells in colons, suggesting that the prototype of NOR was the effective form for its anti-colitis action (Fig. 8a–m).

The correlation between activation of AhR, repression of glycolysis, regulation of NAD^+^/SIRT1/SUV39H1/H3K9me3 signals, induction of Treg cells, and eventual anti-colitis effect of NOR were validated by using CH223191 and HK2 plasmid. CH223191 (10 mg/kg) counteracted NOR (40 mg/kg; i.g.)-induced enhancement of CYP1A1 expression and suppressed expressions of Glut1 and HK2 in colons (Fig. 8a, b). Furthermore, NOR (40 mg/kg; i.g.) remarkably reduced levels of NAD^+^, SIRT1, SUV39H1, and H3K9me3 in colons, which was prevented by CH223191 (10 mg/kg) and HK2 plasmid (10 μg) (Fig. 8c, d). CH223191 (10 mg/kg) and HK2 plasmid (10 μg) also abolished NOR (40 mg/kg; i.g.)-elevated percentages of Treg cells and expressions of Foxp3, IL-10 in colons (Fig. 8e–g). Eventually, the anti-colitis action of NOR (40 mg/kg; i.g.) was counteracted by CH223191 (10 mg/kg) and HK2 plasmid (10 μg), evidenced by detection of body weight change, disease activity index (DAI) scores, colon length, myeloperoxidase (MPO) activity, histological changes, and levels of TNF-α and IL-1β in colons (Fig. 8h–m).

## Discussion

NOR, a natural AhR agonist, yielded excellent amelioration of colitis and accompanied with elevated percentages of Treg cells^17^. However, the deeply mechanisms remain unclear. Under UC condition, colon is the main lesion tissue and undergoes a dramatic hypoxia, which is resulted from an increased metabolic demand of resident and infiltrating inflammatory cells. However, the hypoxic microenvironment of colonic mucosa under UC condition was ignored by many scholars, which is important for Treg differentiation. Therefore, we established the model of Treg differentiation in hypoxia and explored mechanisms from the angle of AhR activation and hypoxia-related miRs and glycolysis. Strikingly, NOR promoted Treg differentiation in hypoxia and normoxia. Furthermore, the minimum effective concentration was lower and the effect was stronger at the same concentration in hypoxia relative to normoxia. In addition, NOR was demonstrated with well AhR activation in CD4^+^ T cells under hypoxic microenvironment and the deeper mechanisms were investigated in the following study.

Hypoxia leads to a variety of biological changes that help cells to adopt to the low oxygen microenvironment. MiRs are small noncoding RNA molecules and intrinsic *miR-31* expression in CD11b^+^ DCs is increased under hypoxic microenvironment^4^; DIM and I3C decrease *miR-31*, *miR-219*, and *miR-490* expressions in draining lymph nodes of mice with delayed-type hypersensitivity^7^. At present, different from the classic AhR agonists, NOR repressed mRNA expression of *miR-31* but *miR-31* mimic exerted little effect on NOR-enhanced Treg polarization. In addition, cellular energy metabolism also appears reprograming in hypoxia and glycolysis holds the dominant position. The glycolysis is stronger in liver cancer cells, HeLa cells, and in *Saccharomyces cerevisiae* under hypoxic microenvironment, and blocking it increases frequencies of Treg cells in vitro and in vivo^8,36,37^. In addition, AhR activation can suppress the process of glycolysis. TCDD disturbs glycolytic pathway by inhibiting the expression of GAPDH, diclofenac disturbs the activity and transcription of LDH in three-spined sticklebacks exposed to hypoxia, and prior polychlorinated biphenyl (PCB) suppresses expressions of glycolytic enzymes in fundulus heteroclitus. Notably, all the actions of TCDD, diclofenac, and PCB are mediated by inhibiting formation of HIF-1α/ARNT complexes^38–40^. Similar to TCDD, NOR significantly repressed glycolysis by reducing the formation of HIF-1α/ARNT complexes and downregulating the expression of HK2, and HK2 plasmid almost completely reversed NOR-promoted Treg differentiation in hypoxia. These findings emphasized the involvement of glycolysis in NOR-enhanced Treg polarization under hypoxic microenvironment.

Increasing evidence indicates that increased glycolysis results in upregulated level of NAD^+^ in cells, as LDH could catalyze the concomitant interconversions of NADH to NAD^+^, thereby increasing cellular NAD^+^ level^22^. In addition, NAD^+^ determines the fate of Treg cells; exogenous NAD^+^ promotes the conversion of Treg into Th17 cells in vitro, in the absence of TGF-β, IL-6, and IL-23, and in the presence of IL-2^23^. Furthermore, NAD^+^ is the substrate and agonist for SIRTs and SIRT1 could deacetylate three novel target sites including K31, K262, and K267 in Foxp3 protein^41^. Treatment with EX-527 promotes expression of Foxp3 during the progress of iTreg differentiation^25^; adoptive transfer of CD4^+^CD25^−^Foxp3^−^ Teff cells lacking SIRT1 to B6/Rag1^–/–^ mice results in higher percentages of Treg cells compared with mice receiving WT Teff cells and subsequent attenuation of colitis^42^. Similar to TCDD, NOR remarkably decreased levels of NAD^+^ and SIRT1 in CD4^+^ T cells under hypoxic condition, which could be prevented by HK2 plasmid, CH223191, and siAhR-3. However, NOR boosted both mRNA and protein expressions of Foxp3 in CD4^+^ T cells, indicating that SIRT1 might not be the final signal molecule for NOR-induced Treg differentiation in hypoxia.

It should be noted that SIRT1 can regulate the protein levels of histone KMTs and histone KDMs. Aguilar-Arnal and colleagues.^26^ indicate that SIRT1 interacts with MLL1 and mediates the deacetylation of MLL1 at two conserved residues, termed K1130 and K1133; Bosch-Presegué and colleagues.^27^ demonstrate that SIRT1 inhibits MDM2 polyubiquitination of lysine 87 in the chromodomain of SUV39H1; Zhao and colleagues^28^ report that SIRT1 knockdown increases EZH2 acetylation, resulting in enhanced stability. Furthermore, expressions of histone KMTs and histone KDMs are altered in hypoxia. MLL1, G9a, SUV39H1, JMJD3, and EZH2 expressions are increased in glioblastoma multiforme, breast cancer cell, and human fetal lung epithelial cells under hypoxic microenvironment, respectively^29–33^. More importantly, they have been demonstrated to participate in Treg differentiation. For example, deficiency of G9A promotes Treg differentiation and results in attenuation of colitis induced by T-cell transfer^43–46^. After performing screening experiments for MLL1, G9a, SUV39H1, JMJD3, and EZH2, we found that NOR only reduced the protein level of SUV39H1 by regulating its ubiquitin-proteasomal degradation and the action was mediated by AhR/glycolysis/SIRT1 signals.

SUV39H1 is the Su (var) 3–9, Enhancer-of-zeste, Trithorax domain-containing histone KMT and participates in H3K9me3 modification. Lacking of SUV39H1 has significant defects in H3K9me3 level and SUV39H1 contributes to facultative heterochromatin formation and gene silencing via elevating H3K9me3 modification^34^. Knockdown of SUV39H1 restores the E-cadherin expression by blocking H3K9me3 modification^47^. Depletion of SUV39H1 or chaetocin (a specific inhibitor of SUV39H1) increases the mRNA expression of BZLF1 in B95-8 cells by reducing the enrichment of H3K9me3 at BZLF1 promoter^48^. Furthermore, the histone modification at promoter, CNS1, 2, and 3 regions can regulate the expression of Foxp3^35^. At present, NOR actually decreased the global level of H3K9me3 and inhibited enrichment of H3K9me3 at − 1,201 to − 1,500 region of Foxp3 promoter; all these effects were reversed by EX-527, HK2 plasmid, CH223191, and siAhR-3. Then, the causal link between activation of AhR, reduction of glycolysis, regulation of NAD^+^/SIRT1/SUV39H1/H3K9me3 signals, induction of Treg cells, and alleviation of colitis by NOR was confirmed in mice with colitis. Finally, the path for NOR-mediated Treg differentiation and anti-colitis action was fully sketched out.

In conclusion, NOR promoted Treg differentiation and then carried out anti-UC action by regulating AhR/glycolysis axis and subsequent NAD^+^/SIRT1/SUV39H1/H3K9me3 signaling pathway.

## Materials and methods

### Chemicals and reagents

NOR (purity > 98%) was isolated and purified from Radix Linderae and the structure was identified by comparison of its spectral data (UV, IR, MS, ^1^H-, and ^13^C-NMR) with the literature data^49^; TCDD was purchased from J&K Chemical (Beijing, China); DSS (molecular weight 36 000–50 000 kDa) was purchased from MP Biomedical (Aurora, USA); CH223191 was purchased from Selleckchem (Houston, USA); FITC-anti-CD4, APC-anti-CD25, PE-anti-Foxp3, and purified anti-mouse CD3/CD28 mAbs were purchased from eBioscience (San Diego, USA); 3-[4,5-dimetylthiazol-2-yl]-2,5-diphenyltetrazolium bromide (MTT) and 2-NBDG were purchased from Invitrogen Corp. (Carlsbad, USA); RPMI 1640 was purchased from Gibco BRL (Grand Island, USA); fetal bovine serum (FBS) was purchased from Sijiqing (Hangzhou, China); antibodies against HK2 and Glut1 were purchased from Sangon Biotech (Shanghai, China); Peroxidase-conjugated secondary antibodies, protein A+G agarose, and cell counting kit-8 (CCK-8) were purchased from Bioworld Technology, Inc. (Georgia, USA); antibodies against AhR, ARNT, HSP90, and Foxp3 were purchased from Santa Cruz Biotechnology (CA, USA); antibodies against SIRT1, SUV39H1, HIF-1α, and H3K9me3 were purchased from Abcam (Cambridge, UK); phenylmethanesulfonyl fluoride (PMSF) and nuclear and cytoplasmic protein extraction kit was purchased from KeyGen Biotech (Nanjing, China); HiScript QRTSuperMix and AceQ qPCR SYBR Green Master Mix were purchased from Vazyme Biotech (Piscataway, USA); mouse IL-1β and TNF-α enzyme-linked immunosorbent assay kits were purchased from Dakewe Biotech (Shenzhen, China); MPO kit was purchased from Jiancheng Biotech (Nanjing, China); ChIP assay kit was purchased from Beyotime Biotechnology (Shanghai, China); siAhR1-3 was purchased from Genechem (Shanghai, China); HK2 plasmid, WT plasmid, mut plasmid, and pGL3-XRE reporter gene plasmid were purchased from Jiman (Shanghai, China); Bulgen-loop miRNA quantitative reverse-transcriptase PCR Primer Sets specific for *miR-31*, *miR-219* and *miR-490*, NC mimic, and *miR-31* mimic were purchased from RiboBio (Guangzhou, China); and Entranster in vivo transfection reagent was purchased from Engree Biosystems Co. (Beijing, China). Other chemical products used were of the analytical grade.

### Animals

Female C57BL/6 mice, weighting 18–22 g (6–8 weeks old), were provided by the Comparative Medicine Center of Yangzhou University (Yangzhou, China). They were housed with free access to food and water under a 12 h light : 12 h dark cycle in plastic cages at 25 ± 2 °C with a relative humidity of 45 ± 10%. The animal experiments were strictly performed in accordance with the Guide for the Care and Use of Laboratory Animals. The protocol was approved by the Animal Ethics Committee of China Pharmaceutical University.

### Induction, drug administration, and assessment of UC

Mice were fed with 2.5% DSS for 7 days and followed by 3 days of drinking water alone. NOR (40 mg/kg) was i.g. or p.r. administered daily for consecutive 10 days; TCDD (25 μg/kg) was intraperitoneally (i.p.) administered only at day 1; CH223191 (10 mg/kg) was i.p. administered daily for consecutive 10 days; HK2 plasmid was mixed with equal volume Entranster in vivo transfection reagent, and p.r. administrated daily for consecutive 10 days. To identify the involvement of AhR and glycolysis in NOR-attenuated colitis, mice were randomly divided into nine groups as follows: normal group, DSS group, NOR (40 mg/kg, i.g.) group, NOR (40 mg/kg, i.g.) +CH223191 (10 mg/kg, i.p.) group, CH223191 (10 mg/kg, i.p.) group, NOR (40 mg/kg, i.g.) + HK2 plasmid (10 μg, p.r.) group, HK2 plasmid (10 μg, p.r.) group, TCDD (25 μg/kg, i.p.) group, and NOR (40 mg/kg, p.r.) group.

Body weight, stool consistency, and the presence of gross blood were observed every day. The DAI was calculated as the mean value of body weight loss, stool consistency, and gross bleeding. In addition, the colons were collected and photographed on day 10. The distal was fixed in 10% formalin for histopathological examination and scores were calculated by assessing the inflammation severity, extent of injury, and crypt damage as previously described^17^.

### Cell culture

CD4^+^ T cells were isolated from mesenteric lymph nodes (MLNs) of C57BL/6 mice and purified with magnetic beads according to the manufacturer’s instructions (Miltenyi Biotech, Cologne, Germany). They were maintained in complete RPMI 1640 medium supplemented with 100 U/ml of streptomycin, 100 U/ml of penicillin, and 10% FBS under hypoxic (supplied with 2% oxygen) or normoxic (supplied with 21% oxygen) microenvironment.

### Cell viability assay

CD4^+^ T cells were seeded into 96-well plates and treated with NOR (0.1, 0.3, 1, 3, 10, 30, 60, 100 μM) in hypoxia or normoxia for 68 h. Subsequently, MTT (5 mg/ml, 20 μl) or CCK-8 solution (10 μl) were added and cells were continuously incubated for an additional 4 h. For MTT assay, the supernatants were removed, formazan crystals were dissolved in 200 μl dimethyl sulfoxide, and optical absorbance at 570 nm was read by a Microplate Reader (Thermo, Waltham, MA, USA). For CCK-8 assay, the supernatants were retained and optical density absorbance at 450 nm was read by a Microplate Reader.

### Flow cytometry

Lymphocytes were harvested from in vitro culture or isolated from MLNs and colonic lamina proprias. The method for lymphocytes isolation was performed as described in our previous study^17^. Subsequently, they were stained with FITC-anti-CD4 and APC-anti-CD25 antibodies for 30 min at 4 °C, followed by fixation and permeabilization for 5 h. Then, they were stained with PE-anti-Foxp3 antibody for another 1 h, washed with fluorescence-activated cell sorting staining buffer, and analyzed by BD FACS Calibur (BD Biosciences, San Jose, USA). All the results were analyzed by using Flowjo 7.6 software (Treestar, Ashland, OR).

### Differentiation of Treg cells

CD4^+^ T cells were treated with anti-CD3/CD28 (2 μg/ml), NOR (1, 3, 10, 30 μM), and TCDD (5 nM) under hypoxic or normoxic condition for 72 h. The frequencies of Treg cells were detected by using flow cytometry.

### Transfection

For stable transfection: CD4^+^ T cells were transfected with lentivirus-mediated siAhR1-3 for 72 h according to the manufacturer’s protocols^50^. For transient transfection: CD4^+^ T cells were transfected with HK2 plasmid, *miR-31* mimic, WT plasmid, and mut plasmid by using Lipofectamine 2000 (Invitrogen Corp.) for 24 h according to the manufacturer’s protocols^51^.

### Quantitative PCR

Total RNA was isolated from cultured cells or colons by TRIzol extraction reagent according to the manufacturer’s instructions (Invitrogen Corp.). Subsequently, RNA (2 µg) was reversed transcribed into cDNA by using HiScript QRTSuperMix. The cDNA template (2 µl) was added to the 20 µl PCR reaction, which contained sequence-specific primers and the AceQ qPCR SYBR Green Master Mix reagent. The cycling conditions included an initial step at 95 °C for 5 min, followed by 40 cycles at 95 °C for 10 s and 55–60 °C for 30 s. The primers were listed in Supplementary Table 1.

### Immunofluorescence

CD4^+^ T cells were treated with anti-CD3/CD28 (2 μg/ml), NOR (1, 3, 10, 30 μM), and TCDD (5 nM) in hypoxia or normoxia for 48 h, fixed with 4% paraformaldehyde for 30 min, and permeabilized with 0.2% Triton-100 for 20 min. Subsequently, they were blocked with 5% bovine serum albumin for 2 h and incubated with anti-Foxp3 antibody (1 : 150) at 4 °C for overnight. After being washed with phosphate-buffered saline (PBS), cells were stained with rhodamine-conjugated affinipure goat anti-mouse IgG antibody (1 : 100) for 2 h. Subsequently, coverslips were stained with 4', 6-diamidino-2-phenylindole for 20 min and images were captured by using Olympus IX53 (Olympus, Tokyo, Japan).

### Cellular uptake of NOR

CD4^+^ T cells were treated with anti-CD3/CD28 (2 μg/ml), NOR (30 μM) in hypoxia for 4 h, and lysed in water by three freeze–thaw cycles followed by centrifugation at 5,000 r.p.m. for 10 min. The supernatants were transferred to the centrifuge tube and 1 ml acetonitrile was added to extract NOR. The extracts were centrifugated at 12 000 r.p.m. for 10 min and the upper organic phase was all carefully transferred to 2 ml EP tube. After being dried for 2 h, 100 μl mobile phase was added into the tube, which were centrifugated at 12 000 r.p.m. for 10 min. Finally, the supernatants were transferred to 2 ml brown glass vials and an aliquot of 5 μl was injected for LC–MS analysis.

An ACQUITY UPLC BEH C18 (2.1 × 100 mm I.D., 1.7 μm, Waters, Milford, MA, USA) column was used for the analyses. The mobile phase composed of A (0.1% formic acid, v/v) and B (acetonitrile) with a gradient elution: 0–5 min, 90–70% A; 5–7 min, 70-0% A; 7–9 min, 0% A; and 10 min, 90% A. The flow rate of the mobile phase was 0.4 ml/min. All data collected in centroid mode were acquired by using Masslynx V4.1 software (Waters); post-acquisition quantitative analysis was performed by using the QuanLynx program (Waters Corp.). The linear range of NOR in plasma was 10–10,000 ng/ml and the limit of quantification for NOR was 10 ng/ml.

### Glucose uptake

CD4^+^ T cells were treated with anti-CD3/CD28 (2 μg/ml), NOR (1, 3, 10, 30 μM), and TCDD (5 nM) in hypoxia or normoxia for 24 h. After starvation for 4 h, cells were supplied with 2-NBDG (500 μM) and intracellular fluorescence intensity was photographed by using Olympus IX53 as the uptake of glucose.

### XRE-luciferase reporter gene

CD4^+^ T cells were transiently transfected with pGL3-XRE reporter gene vector and renilla luciferase vector by using Lipofectamine 2000. After transfection for ~ 24 h, the media was removed and the cells were treated with NOR (1, 3, 10, 30 μM) and TCDD (5 nM) in hypoxia for an additional 24 h. Subsequently, the luciferase reporter activity was measured by using Dual-Luciferase Reporter Assay System (Promega, WI, USA). Briefly, cells were lysed and transferred into 96-well plates. Approximately 100 μl luciferase substrate was added into each well and the absorbance was detected by using a Microplate Reader.

### Western blotting

#### For total protein extraction

Cells or colons were lysed by using NP40 buffer containing 1 mM PMSF on ice for 15 min and centrifuged at 12 000 r.p.m. for 10 min, and then supernatants were collected. For histone extraction: cells or colons were lysed by using stronger RIPA lysis buffer containing 1 mM PMSF on ice for 15 min and centrifugated at 12 000 r.p.m. for 10 min at 4 °C. The supernatants were discarded and precipitation in 200 μl of 0.25 M HCl was resuspended on a rotator at 4 °C for overnight. Then, the cocktails were centrifuged at 12 000 r.p.m. for 10 min at 4 °C and supernatants were neutralized with an appropriate volume of NaOH.

The total protein and histone at equal amount were separated by SDS-polyacrylamide gel electrophoresis (PAGE). Then, they were transferred onto the polyvinylidene difluoride (PVDF) membranes (Millipore, Billerica, MA), which were blocked with 9% (w/v) non-fat milk for 2 h at room temperature. Subsequently, PVDF membranes were incubated with specific primary antibodies at 4 °C for overnight. After being washed for three times, they were further incubated with horseradish peroxidase-conjugated secondary antibody for 2 h at 37 °C. Lastly, the bands were visualized by using ECL plus reagent.

### Co-immunoprecipitation

CD4^+^ T cells were treated with anti-CD3/CD28 (2 μg/ml), NOR (1, 3, 10, 30 μM), and TCDD (5 nM) in hypoxia for 24 h. Subsequently, they were lysed with stronger RIPA lysis buffer for 15 min and centrifuged at 12 000 r.p.m. for 5 min. The supernatants were collected and incubated with 1 μg antibody against AhR or IgG at 4 °C for overnight, followed by the addition of 20 μl protein A/G agarose beads at 4 °C for 4 h. Afterwards, the cocktails were centrifuged at 5,000 r.p.m. for 10 min and immunoprecipitates were washed with stronger RIPA lysis buffer for four times. The immunoprecipitated proteins were separated by SDS-PAGE gel and western blotting was performed with the indicated antibodies.

### Measurement of cellular NAD^+^ level

CD4^+^ T cells were treated with anti-CD3/CD28 (2 μg/ml), NOR (1, 3, 10, 30 μM), and TCDD (5 nM) in hypoxia for 48 h. Subsequently, they were washed by using ice-cold PBS for three times and incubated with NAD^+^ extraction buffer, which were provided by the manufacturer (AAT Bioquest, CA, USA). Then, cells were centrifuged at 1,000 r.p.m. at 4 °C for 10 min and the supernatants were collected. Approximately 50 μl test sample was added into each well and the absorbance was read at 575 ± 5 nm by a Microplate Reader.

### Chromation immunoprecipitation

CD4^+^ T cells were treated with anti-CD3/CD28 (2 μg/ml), NOR (1, 3, 10, 30 μM), and TCDD (5 nM) in hypoxia for 48 h. Subsequently, they were incubated with 1% formaldehyde for 10 min at room temperature for crosslinking. The reaction was quenched with 125 mM glycine and cells were pelleted and washed with ice-cold PBS. The cell pellets were resuspended in SDS lysis buffer supplemented with PMSF. The cocktails were then sonicated (amplitude, 40 w; process time, 6 min; ON time, 4.5 s; OFF time, 9 s) to shear the DNA and extracts were clarified by centrifugation at 12 000 r.p.m. for 10 min at 4 °C. The supernatants were collected and ~ 1% of the total sheared chromatin was set aside and served as Input control. After being precleared by protein A+G Agarose/Salmon Sperm DNA beads, antibody against H3K9me3 was added and incubated with the extracts on a rotator at 4 °C for overnight. No-antibody controls were always included as the negative control. Then, protein A+G Agarose/Salmon Sperm DNA beads were added and the beads containing protein–DNA complexes were collected. Subsequently, they were washed with low-salt wash buffer, high-salt wash buffer, LiCl wash buffer, and TE buffer to remove nonspecific sequences, and eluted in elution buffer (1% SDS, 0.1 M NaHCO_3_). Finally, de-crosslinking was performed with 5 M NaCl and heated at 65 °C for 4 h. The DNA enrichment was established with Q-PCR and the primers used were listed in Supplementary Table 1.

### Statistical analysis

Data were presented as the means ± SEM. Statistical analysis was performed by PASW statistics 19 software (SPSS, Inc., Chicago, IL). Statistical differences were assessed by one-way analysis of variance test. A value of *P* < 0.05 (*P < *0.05) were accepted as a significant difference.

## Electronic supplementary material

Supplementary Figure 1

Supplementary Figure 2

Supplementary Figure 3

Supplementary Figure 4

Supplementary Figure 5

Supplementary Figure 6

Supplementary Figure 7

Supplementary Table 1

supplementary information
