# Supplementary figures and images for: Norisoboldine, a natural AhR agonist, promotes Treg differentiation and attenuates colitis via targeting glycolysis and subsequent NAD+/SIRT1/SUV39H1/H3K9me3 signaling pathway
Source: Cell Death Dis. 2018 Feb 15;9(3):258. doi: 10.1038/s41419-018-0297-3 (PMC5833367; doi:10.1038/s41419-018-0297-3)

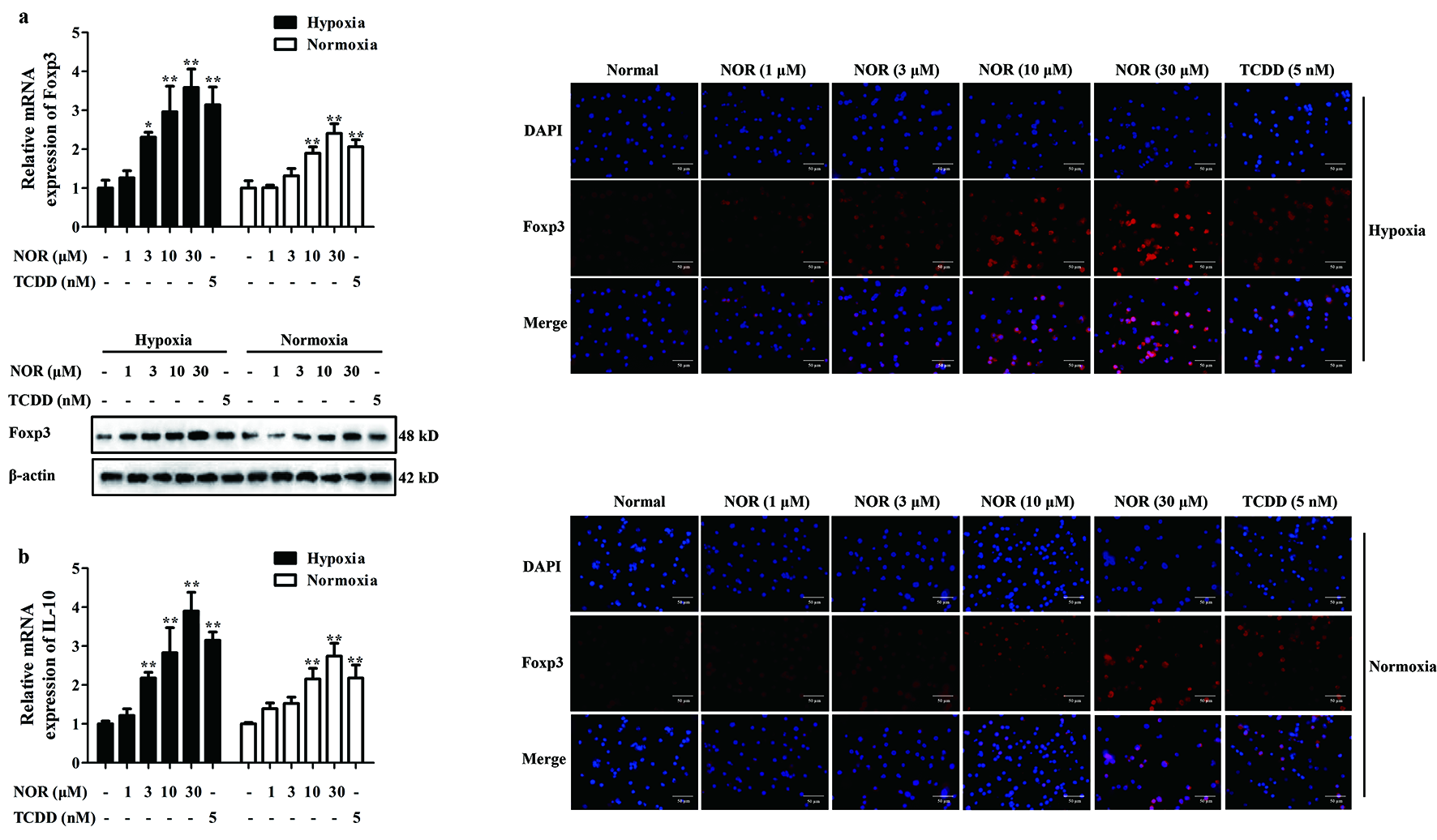

Supplement: Supplementary file 1 — Supplementary Figure 1 [file 41419_2018_297_MOESM1_ESM.tif]

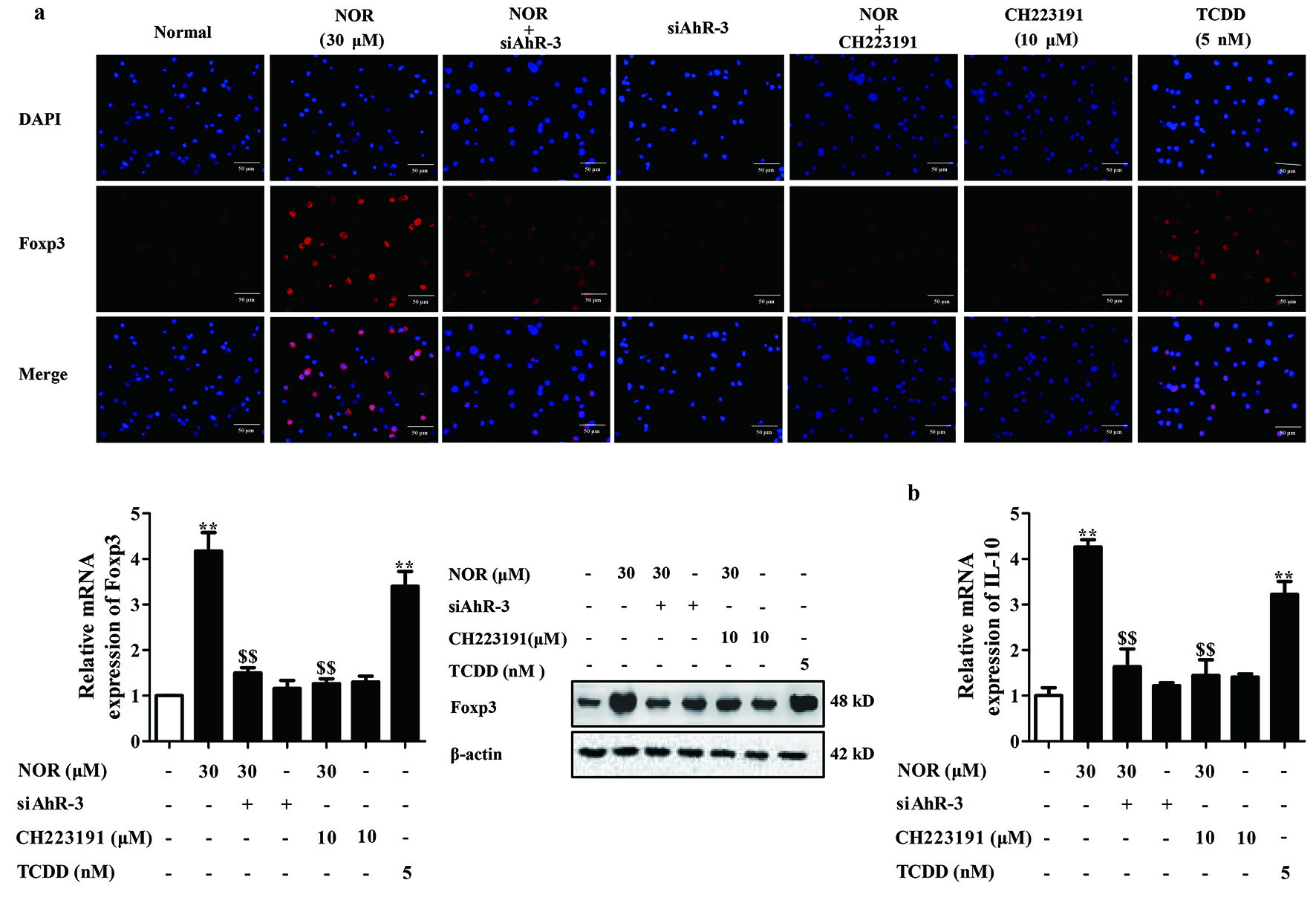

Supplement: Supplementary file 2 — Supplementary Figure 2 [file 41419_2018_297_MOESM2_ESM.tif]

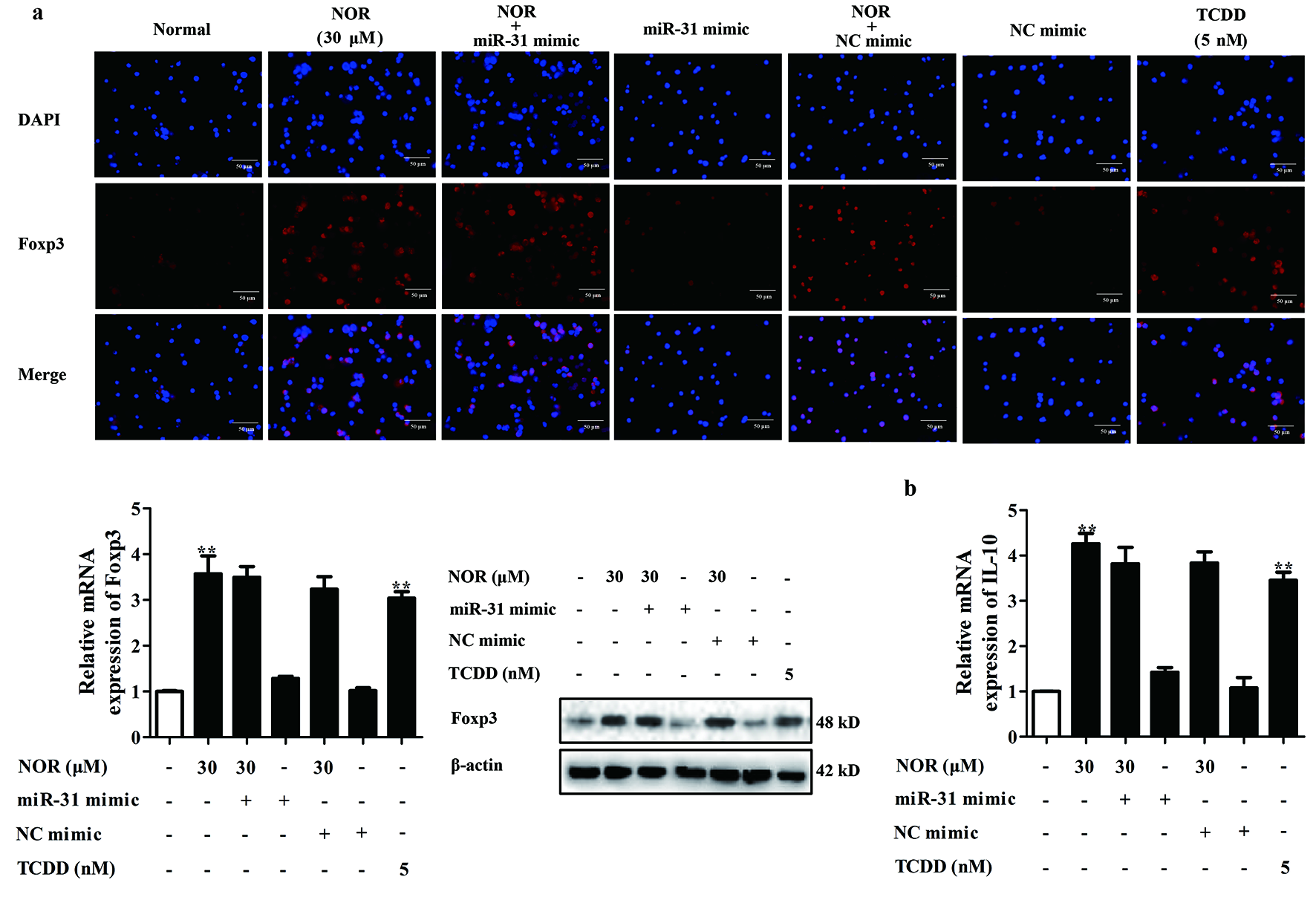

Supplement: Supplementary file 3 — Supplementary Figure 3 [file 41419_2018_297_MOESM3_ESM.tif]

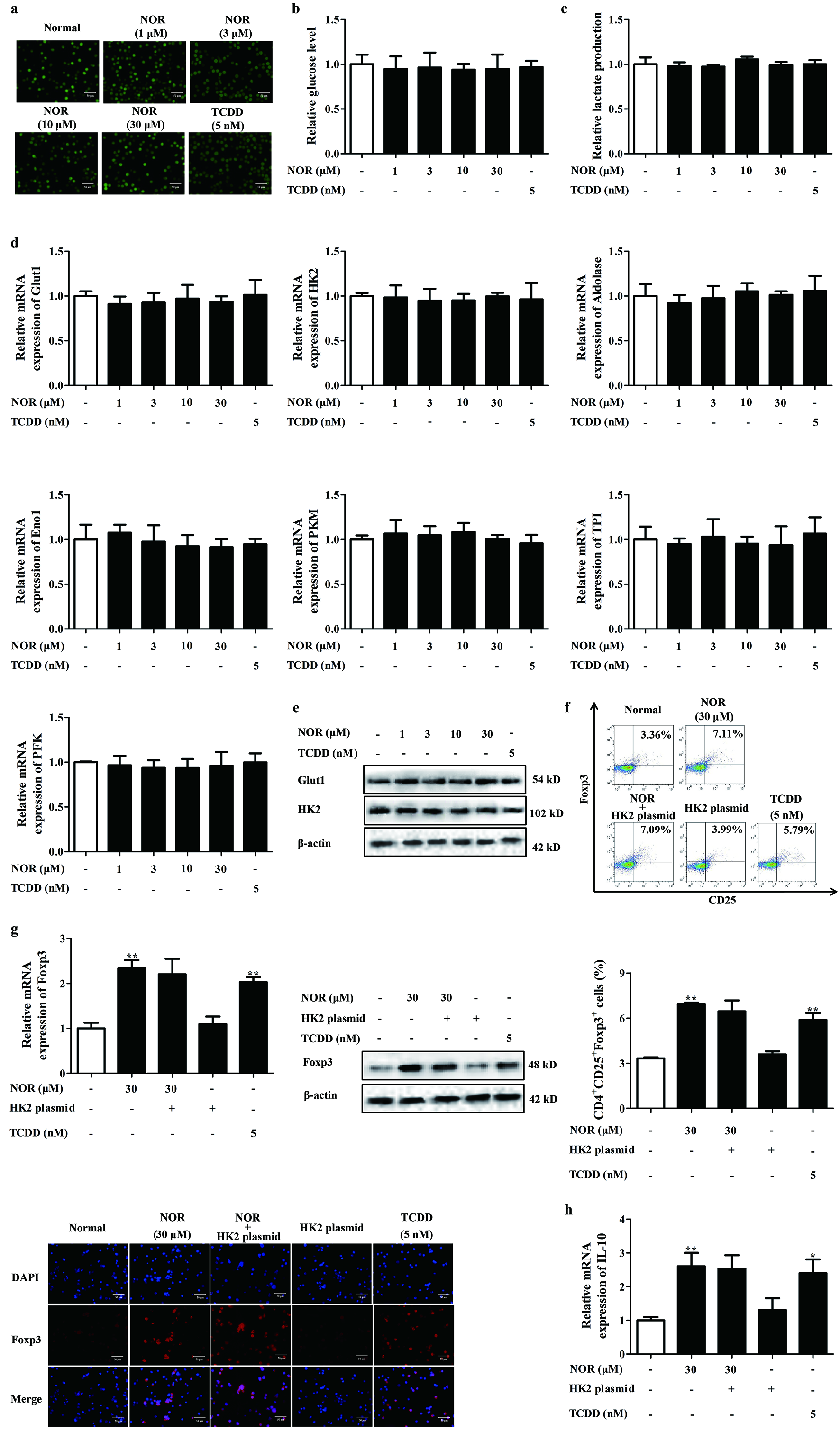

Supplement: Supplementary file 4 — Supplementary Figure 4 [file 41419_2018_297_MOESM4_ESM.tif]

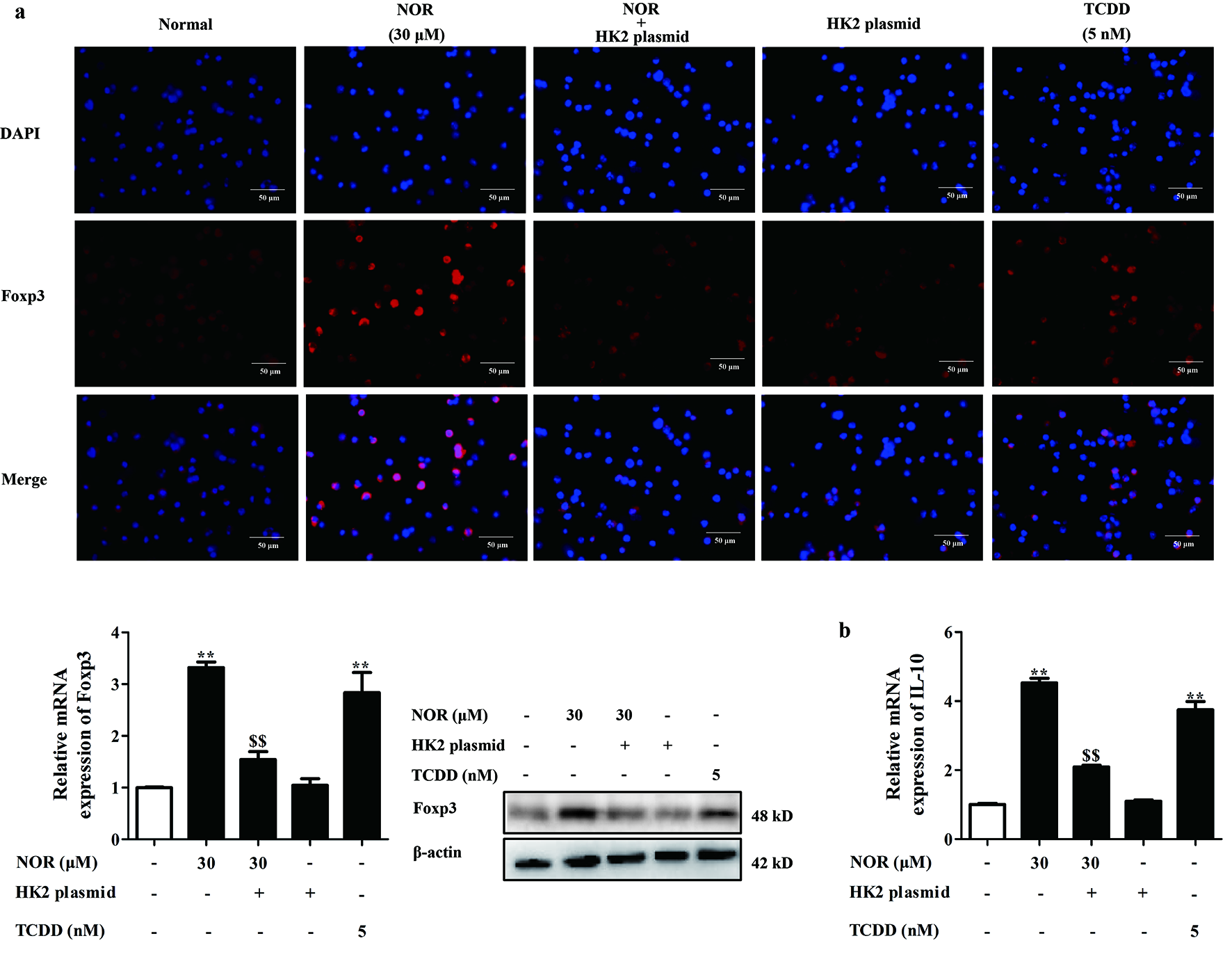

Supplement: Supplementary file 5 — Supplementary Figure 5 [file 41419_2018_297_MOESM5_ESM.tif]

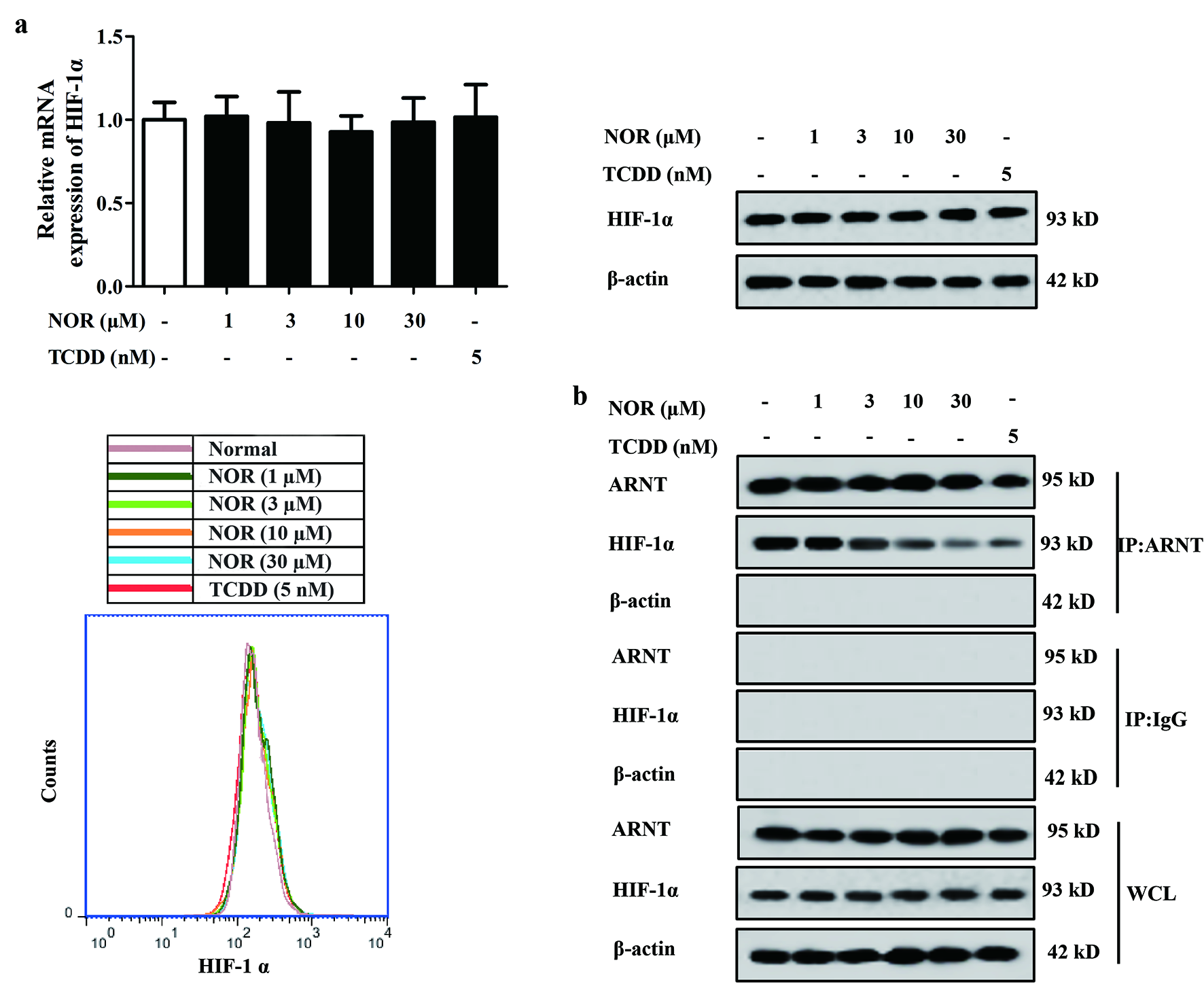

Supplement: Supplementary file 6 — Supplementary Figure 6 [file 41419_2018_297_MOESM6_ESM.tif]

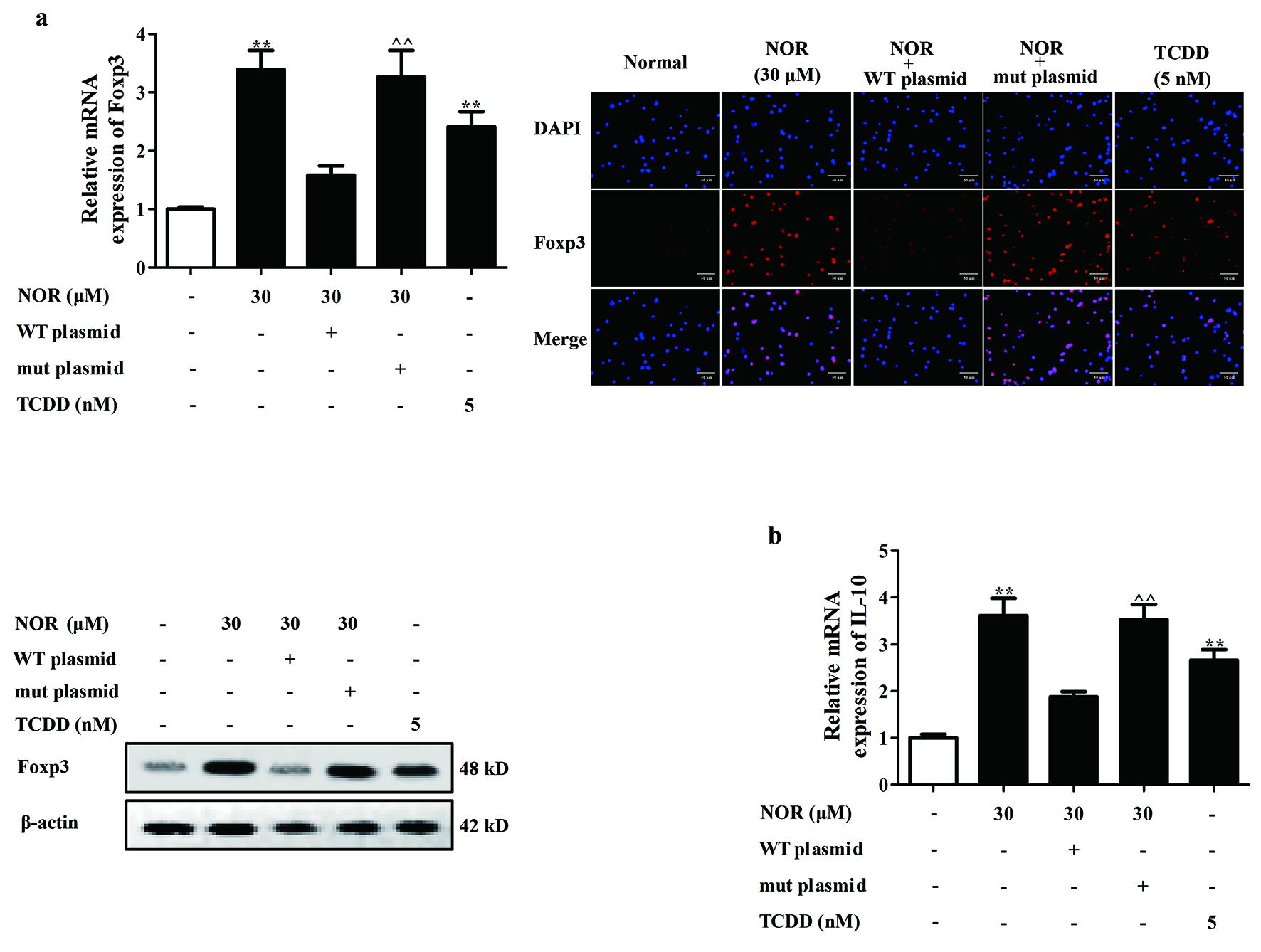

Supplement: Supplementary file 7 — Supplementary Figure 7 [file 41419_2018_297_MOESM7_ESM.tif]
