# Supplementary material for: Norisoboldine, a natural AhR agonist, promotes Treg differentiation and attenuates colitis via targeting glycolysis and subsequent NAD+/SIRT1/SUV39H1/H3K9me3 signaling pathway
Source: Cell Death Dis. 2018 Feb 15;9(3):258. doi: 10.1038/s41419-018-0297-3 (PMC5833367; doi:10.1038/s41419-018-0297-3)
Supplement: Supplementary file 8 — Supplementary Table 1 [file 41419_2018_297_MOESM8_ESM.docx]

**Supplementary Table 1 Primers used in Q-PCR and ChIP assays**

| Primers |  | Sequence (5’-3’) |
| --- | --- | --- |
| GAPDH (mouse) | Forward | GACATTTGAGAAGGGCCACAT |
|  | Reverse | CAAAGAGGTCCAAAACAATCG |
| Foxp3 (mouse) | Forward | GCCCATCCAATAAACTGTGG |
|  | Reverse | GTATCCGCTTTCTCCTGCTG |
| CYP1A1 (mouse) | Forward | GACTTCCAGCCTTCGTGTCA |
|  | Reverse | GGGTTCTTCCCCACAGTCAG |
| TNF-α (mouse) | Forward | AGGCACTCCCCCAAAAGAT |
|  | Reverse | CAGTAGACAGAAGAGCGTGGTG |
| IL-1β (mouse) | Forward | AGTTGACGGACCCCAAAAG |
|  | Reverse | CTTCTCCACAGCCACAATGA |
| IL-10 (mouse) | Forward | GCCTTATCGGAAATGATCCA |
|  | Reverse | AGGGTCTTCAGCTTCTCACC |
| HIF-1α (mouse) | Forward | CAAGCCCTCCAAGTATGAGC |
|  | Reverse | GCCTTAGCAGTGGTCGTTTC |
| Glut1 (mouse) | Forward | GCTTCCTGCTCATCAATCGT |
|  | Reverse | CGACCCTCTTCTTTCATCTCC |
| HK2 (mouse) | Forward | CGACAGCATCATTGTGAAGG |
|  | Reverse | TCCAGTCCACGGTTCTCTCT |
| PKM (mouse) | Forward | TCCCATTCTCTACCGTCCTG |
|  | Reverse | AGTGGCTCCCTTCTTCAGC |
| Aldolase (mouse) | Forward | AAGGCTGCTCCATCAACACT |
|  | Reverse | CACAGACAACACCGCACAC |
| TPI (mouse) | Forward | AGAGAGCCGTGCGTTTGTA |
|  | Reverse | CACTTCTTCCTCCCGTTCAT |
| Eno1 (mouse) | Forward | CAATGATAAGACCCGCTTCA |
|  | Reverse | ATTCTCTGTGCCGTCCATCT |
| PFK (mouse) | Forward | GCCATGCGTAGAGAGGAGTT |
|  | Reverse | TGGTCATGTTGGAGGTTGAA |
| SIRT1 (mouse) | Forward | ACGGTATCTATGCTCGCCTTG |
|  | Reverse | CACAGAGACGGCTGGAACTG |
| SIRT2 (mouse) | Forward | CATCGCGCTTCTTCTCCTG |
|  | Reverse | CTAGTGGTGCCTTGCTGATGA |
| SUV39H1 (mouse) | Forward | GGGAGGAAGAAGTGGAACG |
|  | Reverse | CCCTCCTTCTTCACCTTGC |
| Foxp3 promoter (mouse) | Forward | TCACTTCAGAGCCCAATG |
|  | Reverse | GCCAAACACTTCTCACCC |
| Foxp3 CNS1 (mouse) | Forward | AGACTGTCTGGAACAACCTAGCCT |
|  | Reverse | TGGAGGTACAGAGAGGTTAAGAGCCT |
| Foxp3 CNS2 (mouse) | Forward | ATCTGGCCAAGTTCAGGTTGTGAC |
|  | Reverse | GGGCGTTCCTGTTTGACTGTTTCT |
| Foxp3 CNS3 (mouse) | Forward | TCTCCAGGCTTCAGAGATTCAAGG |
|  | Reverse | ACAGTGGGATGAGGATACATGGCT |
| Foxp3 promoter 1 (mouse) | Forward | ATTTCCGTTTAAGTCTCAT |
|  | Reverse | CGCTCTTCTAATAACCAA |
| Foxp3 promoter 2 (mouse) | Forward | ACTGAGGTTTGGAGCAGAA |
|  | Reverse | GGTTGAACAGAAGAAGGGA |
| Foxp3 promoter 3 (mouse) | Forward | CCTTGCCCTTCTTGGTGAT |
|  | Reverse | TTGTGCTGAGTGCCCTGAC |
| Foxp3 promoter 4 (mouse) | Forward | GGTGCTGGGATTACTGGTAT |
|  | Reverse | CACAAGGCTCTGGGTTGTTA |
| Foxp3 promoter 5 (mouse) | Forward | CAGACCCAATGCTAAGGAC |
|  | Reverse | GAGCCAAACACTTCTCACCC |
| Foxp3 promoter 6 (mouse) | Forward | GCCTTAGCCGTGCCTTGTC |
|  | Reverse | CTGCGTGTCCTCGATTTGG |
| Foxp3 promoter 7 (mouse) | Forward | CCAAGCCATCAGTTCCAGT |
|  | Reverse | TTTGCTCTTTCCCTCATTT |
