# Supplementary material for: Norisoboldine, a natural AhR agonist, promotes Treg differentiation and attenuates colitis via targeting glycolysis and subsequent NAD+/SIRT1/SUV39H1/H3K9me3 signaling pathway
Source: Cell Death Dis. 2018 Feb 15;9(3):258. doi: 10.1038/s41419-018-0297-3 (PMC5833367; doi:10.1038/s41419-018-0297-3)
Supplement: Supplementary file 9 — supplementary information [file 41419_2018_297_MOESM9_ESM.doc]

**Supplementary Figure 1. NOR increases expressions of Foxp3 and IL-10 under hypoxic and normoxic microenvironment.** (a and b) CD4^+^ T cells were cultured with anti-CD3/CD28 (2 µg/mL), NOR (1, 3, 10, 30 μM) and TCDD (5 nM) in hypoxia or normoxia for 48 h. The expression of Foxp3 was analyzed by Q-PCR, western blot and immunofluorescence, respectively. The images were taken at 200 × magnification (Scale bar: 50 μm) (a); the mRNA expression of IL-10 was analyzed by Q-PCR (b). Data were expressed as means ± S.E.M. of three independent experiments. ^*^*P* < 0.05, ^**^*P* < 0.01 *vs.* Normal group.

**Supplementary Figure 2.** **NOR enhances expressions of Foxp3 and IL-10 by activating AhR under hypoxic microenvironment.** (a and b) CD4^+^ T cells were pretreated with CH223191 (10 μM) for 2 h or transfected with siAhR-3, followed with anti-CD3/CD28 (2 µg/mL), NOR (1, 3, 10, 30 μM) and TCDD (5 nM) in hypoxia for 48 h. The expression of Foxp3 was analyzed by immunofluorescence, Q-PCR and western blot, respectively. The images were taken at 200 × magnification (Scale bar: 50 μm) (a); the mRNA expression of IL-10 was analyzed by Q-PCR (b). Data were expressed as means ± S.E.M. of three independent experiments. ^**^*P* < 0.01 *vs.* Normal group; ^$$^*P* < 0.01 *vs.* NOR (30 μM) group.

**Supplementary Figure 3. NOR promotes expressions of Foxp3 and IL-10 in miRs-independent manner under hypoxic microenvironment.** (a and b) CD4^+^ T cells were transfected with miR-31 or NC mimic, followed with anti-CD3/CD28 (2 µg/mL), NOR (1, 3, 10, 30 μM) and TCDD (5 nM) in hypoxia for 48 h. The expression of Foxp3 was analyzed by immunofluorescence, Q-PCR and western blot, respectively. The images were taken at 200 × magnification (Scale bar: 50 μm) (a); the mRNA expression of IL-10 was analyzed by Q-PCR (b). Data were expressed as means ± S.E.M. of three independent experiments. ^**^*P* < 0.01 *vs.* Normal group.

**Supplementary Figure 4. NOR can not influence the process of glycolysis under normoxic microenvironment.** (a-e) CD4^+^ T cells were cultured with anti-CD3/CD28 (2 µg/mL), NOR (1, 3, 10, 30 μM) and TCDD (5 nM) in normoxia for 24 h. Glucose uptake was analyzed by immunofluorescence, and the images were taken at 200 × magnification (Scale bar: 50 μm) (a); glucose consumption was analyzed by kits (b); lactate production was analyzed by kits (c); mRNA expressions of Glut1, HK2, Aldolase, Eno1, PKM, TPI and PFK were analyzed by Q-PCR (d); protein levels of Glut1 and HK2 were analyzed by western blot (e). (f-h) CD4^+^ T cells were transfected with HK2 plasmid, followed with incubation of anti-CD3/CD28 (2 µg/mL), NOR (30 μM) and TCDD (5 nM) in normoxia for indicated time intervals. At 72 h, frequencies of Treg cells were analyzed by flow cytometry (f); at 48 h, mRNA and protein levels of Foxp3 were analyzed by Q-PCR, western blot and immunofluorescence, respectively. The images were taken at 200 × magnification (Scale bar: 50 μm) (g); at 48 h, mRNA expression of IL-10 was analyzed by Q-PCR (h). Data were expressed as means ± S.E.M. of three independent experiments. ^*^*P* < 0.05, ^**^*P* < 0.01 *vs.* Normal group.

**Supplementary Figure 5. NOR induces expressions of Foxp3 and IL-10 in glycolysis-dependent manner under hypoxic microenvironment.** (a and b) CD4^+^ T cells were transfected with HK2 plasmid, followed with anti-CD3/CD28 (2 µg/mL), NOR (1, 3, 10, 30 μM) and TCDD (5 nM) in hypoxia for 48 h. The expression of Foxp3 was analyzed by immunofluorescence, Q-PCR and western blot, respectively. The images were taken at 200 × magnification (Scale bar: 50 μm) (a); the mRNA expression of IL-10 was analyzed by Q-PCR (b). Data were expressed as means ± S.E.M. of three independent experiments. ^**^*P* < 0.01 *vs.* Normal group; ^$$^*P* < 0.01 *vs.* NOR (30 μM) group.

**Supplementary Figure 6.** **NOR inhibits the formation of HIF-1α/ARNT complexes** **under hypoxic microenvironment.** (a and b) CD4^+^ T cells were cultured with anti-CD3/CD28 (2 µg/mL), NOR (1, 3, 10, 30 μM) and TCDD (5 nM) in hypoxia for 24 h. The expression of HIF-1α was analyzed by Q-PCR, western blot and flow cytometry, respectively (a); the formation of HIF-1α/ARNT complexes was analyzed by Co-immunoprecipitation (b). Data were expressed as means ± S.E.M. of three independent experiments.

**Supplementary Figure 7. H3K9me3 modification plays an important role in NOR-induced expressions of Foxp3 and IL-10 under hypoxic microenvironment.** (a and b) CD4^+^ T cells were transfected with bacterial-contained wild type Foxp3 promoter (WT) or -1201 to -1500 region deletion mutant (mut) plasmid, followed with incubation of anti-CD3/CD28 (2 µg/mL), NOR (30 μM) and TCDD (5 nM) in hypoxia for 48 h. The expression of Foxp3 was analyzed by Q-PCR, immunofluorescence and western blot, respectively. The images were taken at 200 × magnification (Scale bar: 50 μm) (a); the mRNA expression of IL-10 was analyzed by Q-PCR (b). Data were expressed as means ± S.E.M. of three independent experiments. ^**^*P* < 0.01 *vs.* Normal group; ^^^^*P* < 0.01 *vs.* NOR+WT plasmid group.
